# Gene signatures of palmitoylation and fatty acid metabolism predict prognosis and immunotherapy response in breast cancer patients by machine learning, single-cell analysis, and experimental validation

**DOI:** 10.3389/fphar.2026.1856591

**Published:** 2026-08-07

**Authors:** Zhun Zhang, Ying Fu, Yusong Zhou, Xinyu Zhou, Wei Liu, Mengjie Tang, Wei Li

**Affiliations:** 1 Department of Breast and Thyroid Surgery, The Third Xiangya Hospital, Central South University, Changsha, China; 2 Department of Nuclear Medicine, Hunan Cancer Hospital, The Affiliated Cancer Hospital of Xiangya School of Medicine, Central South University, Changsha, China; 3 Department of Pharmacy, The Third Xiangya Hospital, Central South University, Changsha, China; 4 Xiangya School of Pharmacy, Central South University, Changsha, China; 5 Department of Pathology, Hunan Cancer Hospital, The Affiliated Cancer Hospital of Xiangya School of Medicine, Changsha, China

**Keywords:** breast cancer, fatty acid metabolism, G6PD, immunotherapy, palmitoylation, prognosis

## Abstract

**Background:**

Palmitoylation and dysregulated fatty acid metabolism are key components of tumor metabolic reprogramming and play important roles in the initiation, progression, and immune regulation of breast cancer (BRCA). However, their systemic molecular characteristics and clinical predictive value remain incompletely understood.

**Methods:**

This study integrated transcriptomic and clinical data from The Cancer Genome Atlas, GTEx, and Gene Expression Omnibus databases to identify genes associated with fatty acid metabolism and palmitoylation that are aberrantly expressed in tumors. Using Cox and LASSO regression analyses, we constructed a prognostic risk model to stratify patients into distinct risk groups and evaluated differences in survival outcomes, immune profiles, and predicted therapeutic responses. Furthermore, we performed additional analyses, including pan-cancer analysis, tumor microenvironment characterization, drug sensitivity prediction, single-cell RNA sequencing analysis, and *in vitro* and *in vivo* functional experiments, to investigate the potential biological and clinical relevance of these findings.

**Results:**

This study identified a set of genes associated with palmitoylation and fatty acid metabolism that are significantly linked to BRCA prognosis, and constructed a prognostic risk model to stratify patients into distinct risk groups. Significant differences were observed between the high- and low-risk groups regarding overall survival, immune cell infiltration, immune checkpoint expression, and predicted immunotherapy efficacy. Further analysis identified G6PD as a key gene associated with poor prognosis. Epithelial cells with high G6PD expression exhibited enhanced activity in palmitoylation and fatty acid metabolism pathways, accompanied by altered intercellular communication within the TME. Knockdown of G6PD inhibited the proliferation and migration of BRCA cells and suppressed tumor growth *in vivo*. Potential interactions between G6PD and candidate compounds suggest its therapeutic potential.

**Conclusion:**

This study indicates that gene signatures associated with palmitoylation and fatty acid metabolism may be relevant to prognostic stratification and the prediction of immunotherapy efficacy in BRCA. Furthermore, the study identifies G6PD as a key metabolic gene linked to tumor progression; this finding not only offers potential insights into the molecular mechanisms of BRCA but also supports further exploration of metabolism-related therapeutic strategies.

## 1 Introduction

BRCA remains one of the leading malignancies in women worldwide in terms of both incidence and mortality, and its initiation and progression are influenced by multiple factors, including genetic alterations, the TME, and metabolic reprogramming (Zhang et al., 2021a; Wang et al., 2025; Malla et al., 2025; Zhao et al., 2025; Wu et al., 2025). Despite advances in molecular subtyping and therapeutic strategies, substantial heterogeneity in prognosis and treatment response persists among BRCA patients, particularly in immunotherapy and systemic treatment, where variable efficacy is commonly observed (Dvir et al., 2024; Kundu et al., 2024). These observations indicate that traditional clinicopathological features and single-gene biomarkers are insufficient to fully capture tumor complexity. Therefore, there is a need to identify robust molecular signatures with potential prognostic and predictive value from emerging biological perspectives.

Tumor metabolic reprogramming is a hallmark of malignancy (Li et al., 2026; Bian et al., 2025), providing energy and biosynthetic precursors for tumor growth while also modulating signaling pathways and intercellular communication to influence tumor progression and immune responses (Lin et al., 2024; Mao et al., 2024). Among these metabolic processes, fatty acid metabolism is particularly active in BRCA (He et al., 2023). Fatty acids function as energy substrates, structural components of cellular membranes, and signaling molecules, thereby contributing to tumor cell proliferation, invasion, and therapeutic resistance (Currie et al., 2013). Previous studies have reported that dysregulated fatty acid metabolism is closely associated with aggressive tumor phenotypes and poor prognosis in BRCA (Karmokar and Moniri, 2022; Zhang et al., 2021b); however, its comprehensive molecular characteristics and clinical relevance remain insufficiently understood.

Palmitoylation is a reversible lipid post-translational modification that regulates protein localization, stability, and function through the covalent attachment of fatty acids to target proteins (Ko and Dixon, 2018). Emerging evidence indicates that palmitoylation plays important roles in multiple tumor-related signaling pathways and is closely associated with tumor growth and metastasis (Li et al., 2023). In addition, palmitoylation is functionally interconnected with fatty acid metabolism, and both processes cooperatively contribute to tumor metabolic adaptation and signal transduction (Leishman et al., 2024). However, in BRCA, whether palmitoylation and fatty acid metabolism form a coordinated regulatory network that influences patient prognosis and immunotherapy response remains insufficiently explored. With the rapid development of high-throughput sequencing and bioinformatics approaches, multi-gene metabolic signatures have become an important strategy for characterizing tumor heterogeneity and supporting precision oncology (Vanella et al., 2022; Tian et al., 2024). Moreover, single-cell transcriptomic technologies provide opportunities to resolve metabolic states and tumor microenvironment interactions at single-cell resolution, offering new insights into tumor metabolic regulation and immune modulation (Liu et al., 2024).

Against this backdrop, the present study integrates transcriptomic data from multiple databases with clinical information to systematically identify BRCA-associated genes from the perspectives of fatty acid metabolism and palmitoylation, and to construct a prognostic risk model. We further evaluated the ability of this model to stratify patients based on survival outcomes and predicted immunotherapy response. In addition, by combining single-cell transcriptomic analysis with *in vitro* and *in vivo* functional experiments, we investigated the biological roles of key metabolic genes in BRCA progression. Overall, this study aims to provide a theoretical framework based on metabolic reprogramming for improving the molecular stratification of BRCA patients and to offer potential insights into metabolism-related therapeutic strategies.

## 2 Materials and methods

### 2.1 Data acquisition and preprocessing

RNA sequencing (RNA-seq) expression data, genomic mutation data, and corresponding clinical information for BRCA were downloaded from The Cancer Genome Atlas (TCGA) database (1,118 tumor samples and 113 normal samples) and the Gene Expression Omnibus (GEO) database (GSE58812, 107 tumor samples). Gene expression data for normal breast tissues were obtained from the Genotype-Tissue Expression (GTEx) database (https://gtexportal.org; accessed on 7 February 2025), including 60 normal samples. Raw data from different datasets were processed using Perl (version 5.30) to standardize gene identifiers and sample annotations, and merged into a unified expression matrix for downstream analyses. Batch effect correction and data integration between TCGA and GTEx, as well as between TCGA and GEO datasets, were performed prior to further analysis. The “limma” R package was subsequently used for differential expression analysis.

### 2.2 Differential expression analysis and extraction of gene expression levels

The “limma” R package was used to perform differential expression analysis between BRCA samples and normal tissues from the TCGA and GTEx databases, thereby identifying differentially expressed genes (DEGs). Fatty acid metabolism-related gene sets were obtained from the GSEA database, including HALLMARK_FATTY_ACID_METABOLISM, KEGG_FATTY_ACID_METABOLISM, REACTOME_FATTY_ACID_METABOLISM, KEGG_GLYCEROLIPID_METABOLISM, GOBP_FATTY_ACID_ALPHA_OXIDATION, and KEGG_PPAR_SIGNALING_PATHWAY. Palmitoylation-related genes were retrieved from the SwissPalm database. The intersection of fatty acid metabolism-related genes, palmitoylation-related genes, and DEGs was identified using the “VennDiagram” R package and built-in intersect functions, yielding a set of differentially expressed palmitoylation- and fatty acid metabolism-related genes (PAFARGs) in BRCA. Finally, the expression profiles of PAFARGs were extracted from the integrated TCGA and GEO datasets for subsequent analyses. To investigate biological network integration for gene prioritization and to predict the functional roles of these prognostic genes, the GeneMANIA prediction server (http://www.genemania.org; accessed on 28 May 2025) was utilized in this study.

### 2.3 Screening of prognostic genes

Using the R packages “limma”, “survival” and “survminer”, and integrating gene expression data files for PAFARGs with corresponding clinical data files, univariate Cox regression analysis was performed to screen for prognostic genes with a *p*-value <0.05, followed by the generation of a forest plot.

### 2.4 Pan-cancer analysis of prognosis-related palmitoylation and fatty acid metabolism genes

The Gene Set Cancer Analysis (GSCA) platform is an integrated resource for the gene set-based cancer analysis of genomics, pharmacogenomics, and immunogenomics (Subramanian et al., 2005) (http://bioinfo.life.hust.edu.cn/GSCA; accessed 1 June 2025). The GSCA data platform was utilized to conduct a pan-cancer analysis of the identified prognostic genes across 33 distinct cancer types, focusing on the following aspects: the association between GSVA scores and survival outcomes (including OS, PFS, DSS, and DFI); the correlation between GSVA scores and pathway activity; and the correlation between gene expression levels and drug sensitivity data from both the GDSC and CTRP databases.

### 2.5 Unsupervised clustering analysis and functional enrichment analysis

The R package “ConsensusClusterPlus” was employed to perform consensus clustering analysis on the expression data of the prognostic genes identified in the initial screening phase. To ensure the accuracy and stability of the classification, the resampling iteration count was set to 50. The optimal number of clusters was determined based on the clustering results, and t-distributed stochastic neighbor embedding (t-SNE) was utilized to visualize the distribution of the identified clusters. Subsequently, the R packages “survival” and “survminer” were used in conjunction with clinical data to conduct survival analyses for each of the identified clusters. To elucidate the biological functional differences between the identified subtypes and to validate the biological plausibility of the clustering results, the R packages “clusterProfiler” and “org.Hs.eg.db” were used to perform Gene Ontology (GO) and Kyoto Encyclopedia of Genes and Genomes (KEGG) functional enrichment analyses on the differentially expressed genes identified between the clusters. Finally, the R packages “ggplot2” and “enrichplot” were utilized for data visualization, generating bubble plots to illustrate the enrichment results.

### 2.6 Construction and Validation of prognostic models related to PAFARGs

Based on genes identified as having prognostic value through univariate Cox regression analysis, LASSO regression analysis was employed to identify key genes and develop a prognostic signature. In this analysis, random stratified sampling was performed on the gene expression and survival data using the R package “caret”, allocating 50% of the samples to a training set and the remaining 50% to a test set. Within the training set, LASSO regression was used to screen key genes associated with survival, and a multivariate Cox proportional hazards model was constructed to calculate a risk score for all samples. Using the median risk score of the training set as a threshold, samples were stratified into a high-risk group and a low-risk group. The test set was used to validate the model’s predictive capability on independent data, thereby ensuring the reproducibility of the results. If the model derived from the training set demonstrated insufficient prognostic performance in the test set, the grouping and training process was repeated. The R packages “survival”, “survminer” and “timeROC” were utilized to generate survival curves for the high- and low-risk groups, plot 1-, 3-, and 5-year Receiver Operating Characteristic (ROC) curves, and construct clinical ROC curves by incorporating clinical data. Additionally, the R package “pheatmap” was used to visualize patient risk scores, risk heatmaps, and survival status plots. Furthermore, univariate and multivariate analyses incorporating clinical data were conducted to investigate the independent prognostic significance of the risk model.

### 2.7 Further screening of prognostic genes

Based on differences in gene expression levels among patient samples, genes were stratified into high- and low-expression groups. By integrating clinical information, Kaplan-Meier (K-M) survival analysis was performed to compare survival differences between these two groups; survival curves were plotted for genes with a *p*-value <0.05. These survival curves were then analyzed in conjunction with the differential expression levels of the key genes in BRCA tumor tissues versus adjacent normal tissues to further refine the selection of prognostic key genes. The screened genes underwent subsequent univariate and multivariate Cox regression analyses; those prognostic genes with a *p*-value <0.05 were identified as the target genes possessing independent prognostic significance.

### 2.8 Construction of nomograms and development of mutational signatures

By integrating clinical data, the R package “regplot” was used to construct a nomogram, combining variables such as gender, histological grade, age, and risk score to predict survival outcomes. Furthermore, calibration curves were plotted to evaluate the accuracy of the nomogram in predicting the probabilities of 1-year, 3-year, and 5-year overall survival (OS). The frequencies of copy number variations in BRCA samples were obtained from the TCGA database; mutation data were stratified into high- and low-risk groups based on risk scores. The R package “maftools” was then used to visualize the top 15 most frequently mutated genes in both the high- and low-risk groups using waterfall plots.

### 2.9 Analysis of the tumor immune microenvironment and prediction of immunotherapy response

To enhance our understanding of the immunological landscape in BRCA patients, immune infiltration analysis (CIBERSORT) was employed to estimate the proportions of various immune cells within the TME. We analyzed the correlations between the risk scores derived from hub genes and the levels of immune cell infiltration, visualizing the results using bubble plots. The R package “estimate” was employed to calculate TME scores for BRCA samples, while the “limma” package was used to perform differential expression analysis between high- and low-risk groups based on risk scores, with results visualized using violin plots. Additionally, to further elucidate the immunological characteristics associated with the hub gene-derived risk scores, this study retrieved a panel of 47 immune checkpoint genes from previously published literature. We assessed their correlations with the hub genes and the risk scores, subsequently generating a correlation heatmap.

### 2.10 Drug sensitivity analysis and molecular docking

To further investigate the clinical significance of G6PD, we performed a correlation analysis between G6PD expression levels and drug sensitivity using the CTRP and PRISM databases, accessible via the BEST website (https://rookieutopia.hiplot.com.cn/app_direct/BEST/). The objective of this analysis was to screen for potential anti-BRCA drugs associated with G6PD. Subsequently, we selected drugs that demonstrated a negative correlation with high G6PD expression in the correlation analysis and subjected them to molecular docking studies. The names, molecular weights, and 3D structures of these small-molecule drugs were retrieved from the PubChem database. Finally, we downloaded the 3D protein structure corresponding to the G6PD gene from the RCSB PDB database (http://www.rcsb.org/; accessed on 6 January 2026). AutoDock Vina software (https://vina.scripps.edu/; accessed 6 January 2026) was used to prepare the ligands and proteins for the molecular docking process. Affinity values (kcal/mol) were used to indicate the binding strength between the two entities; lower affinity values (kcal/mol) suggest a more stable binding between the ligand and the receptor. The results were visualized using PyMOL (version 3.0).

### 2.11 Single-cell analysis

Single-cell RNA sequencing data were obtained from the Gene Expression Omnibus (GEO) database (Accession No. GSE161529; https://www.ncbi.nlm.nih.gov/geo/query/acc.cgi?acc=GSE161529). All analyses were performed using the “Scanpy” package in Python. After importing the raw gene expression matrix, the data underwent preprocessing according to a standard workflow: cells with fewer than 200 detected genes or with a mitochondrial gene content exceeding 20% ​​were excluded. Following the Scanpy preprocessing pipeline, gene expression data were normalized, log-transformed, and corrected for technical confounding factors (such as total counts and mitochondrial gene content), culminating in a final normalization step. Dimensionality reduction and batch effect correction were performed using the PCA and BBKNN algorithms, respectively; data visualization was achieved using the UMAP algorithm, while clustering analysis was conducted using the Leiden algorithm. Cell types were identified based on canonical marker genes, publicly available annotation information, and inferred functional states. To further investigate intercellular communication networks within the TME, we also utilized the CellChat tool to perform relevant analyses.

### 2.12 Tissue samples and cell sources

The human breast tissue samples utilized in this study were collected at the Third Xiangya Hospital of Central South University. All subjects provided written informed consent prior to surgery, and the study protocol was reviewed and approved by the Ethics Committee of the Third Xiangya Hospital of Central South University. The collected tissue samples were primarily used for immunohistochemistry (IHC) analysis to assess the expression levels of G6PD in BRCA tissues and their corresponding adjacent normal tissues. The normal breast epithelial cell line MCF-10A, as well as the BRCA cell lines BT-549 and MDA-MB-231, were purchased from the American Type Culture Collection (ATCC). All cell lines were cultured in Dulbecco’s Modified Eagle Medium (DMEM) supplemented with 10% fetal bovine serum (FBS) and were maintained in a humidified incubator at 37 °C with 5% CO_2_.

### 2.13 MTT assay

Following the transduction of these cells with the G6PD plasmid and a control plasmid, the cells were subsequently seeded into 96-well plates at a density of 6 × 10^3^ cells per well. After continuing the culture for 72 h, 50 µL of MTT solution (at a concentration of 2 mg/mL) was added to each well, followed by incubation in an incubator for 4 h. After removing the culture medium, 150 µL of DMSO was added to each well; the plates were then gently shaken for 10 min, and finally, the absorbance values were measured at a wavelength of 490 nm.

### 2.14 Colony formation assay

Following the transduction of these cells with the G6PD plasmid and a control plasmid, the cells were seeded into 6-well plates at a density of 1,000 cells per well and cultured in an incubator, with the culture medium replaced every three to 4 days. After 12–14 days, when visible and well-defined colonies had formed, the culture was terminated. 1 mL of 10% paraformaldehyde fixative was added to each well to fix the cells for 20 min, followed by two washes with PBS. Finally, the cells were stained with 0.1% crystal violet for 30 min, washed twice with PBS, allowed to air-dry, and photographed; ImageJ software was then used to quantify the number of formed colonies.

### 2.15 Wound healing assay

Following the transduction of these cells with the G6PD plasmid and a control plasmid, the cells were seeded into 12-well plates at a density of 3 × 10^5^ cells per well. After 24 h, when the cells had formed a confluent monolayer, a linear wound was generated by gently scraping the cell layer with a 200 μL pipette tip. The cells were then washed twice with PBS and subsequently cultured in serum-free medium. Images of the same location within the wound area were captured using a microscope at 0 h and 24 h. ImageJ software was used to measure the width of the scratch, thereby calculating the cell migration rate.

### 2.16 Transwell migration assay

Following the transduction of these cells with the G6PD plasmid and a control plasmid, the cells were seeded into the upper chamber of Transwell inserts at a density of 20,000 cells per well (suspended in 200 µL of serum-free medium per well), while 600 µL of complete culture medium was added to the lower chamber. Continue incubation in an incubator for 24 h. Remove the Transwell inserts, fix with 4% paraformaldehyde for 20 min, and wash twice with PBS. Subsequently, stain with 0.1% crystal violet for 20 min, wash twice with PBS, and use a cotton swab to remove the cells located on the upper surface of the membrane that have not migrated into the lower chamber. Observe under a microscope and capture images; finally, use ImageJ to count the cells and calculate the cell migration rate.

### 2.17 Western blot

Following transduction of the cells with either the G6PD plasmid or a control plasmid, the cells were seeded into 6-well plates at a density of 3 × 10^5^ cells per well and cultured for an additional 48 h. Total protein was then extracted using RIPA lysis buffer and quantified using a BCA protein assay kit. After denaturation, 30 µg of the extracted protein was separated via SDS-PAGE and subsequently transferred onto a PVDF membrane. The membrane was then blocked with 5% non-fat dry milk for 2 h. Subsequently, the membrane was incubated overnight at 4 °C with a primary antibody specific to the target protein, followed by a 1-h incubation with a horseradish peroxidase (HRP)-conjugated secondary antibody. Antigen-antibody interactions were visualized using a ChemiDoc imaging system. Finally, ImageJ software was used to quantify the grayscale intensity of the bands and calculate the relative protein expression levels.

### 2.18 Quantitative real-time PCR

Following transduction of the cells with either the G6PD plasmid or a control plasmid, the cells were seeded into 12-well plates at a density of 3 × 10^5^ cells per well and cultured for an additional 24 h. Total RNA was then extracted using an RNeasy Mini Kit (Qiagen, Beijing, China). The total RNA was reverse-transcribed into cDNA using a High-Capacity cDNA Reverse Transcription Kit (Thermo Fisher Scientific, Shanghai, China). The PCR reactions were performed according to the manufacturer’s instructions using TaqMan probes specific for human G6PD and GAPDH genes, which were synthesized by Sangon Biotech (Shanghai) Co., Ltd.

### 2.19 Immunohistochemistry

Harvested tissue samples were fixed in 4% paraformaldehyde, embedded in paraffin, and sectioned into 6 µm-thick slices. The sections were then deparaffinized, rehydrated, and subjected to immunohistochemical staining. To expose antigens and inactivate endogenous peroxidase activity, the rehydrated sections were treated with Tris-EDTA buffer (containing 10 mM Tris-HCl and 1 mM EDTA) and subsequently heated in a pressure cooker until boiling for 5 min. After washing the sections three times, they were incubated with bovine serum albumin (BSA) at room temperature for 30 min, followed by overnight incubation with the primary antibody of interest at 4 °C. The following day, the sections were incubated with the secondary antibody at room temperature for 1 h. Subsequently, the sections were stained with hematoxylin, dehydrated, and mounted using neutral resin; they were then observed and photographed under a microscope.

### 2.20 Nude mouse xenograft model

The nude mice used in this study were 4-week-old female BALB/c nude mice, purchased from Skajingda Biotechnology Co., Ltd., and housed in a specific pathogen-free (SPF) animal facility. The experimental environment was maintained at a temperature of 22 °C–26 °C with approximately 55% humidity and a 12-h light/dark cycle. This study was approved by the Institutional Animal Care and Use Committee of Central South University. A maximum of five mice were housed per cage; to avoid experimental bias, the mice were randomly assigned to different experimental groups. One week after the mice had acclimated to the environment, they were subcutaneously injected with a suspension containing 2 × 10^6^ MDA-MB-231-shCtrl cells or MDA-MB-231-shG6PD cells. Once the tumor volume reached 70–100 mm^3^, tumor size (calculated using the formula: 1/2 × length × width^2^) and mouse body weight were measured every 2 days. After 15 days, the tumors were excised, photographed, and weighed. Half of each tumor was collected for immunohistochemical staining.

### 2.21 Assessment of CD8^+^ T-Cell cytotoxicity

CD8^+^ T cells were isolated according to previously published literature (Shen et al., 2022). After tumor cells were transfected with either the G6PD plasmid or a control plasmid, they were seeded into 96-well plates at a density of 1 × 10^4^ cells per well and cultured for an additional 12 h. Subsequently, CD8^+^ T cells were added to the 96-well plates, and the co-culture was incubated for 48 h. The old culture medium was then removed, and the cells were washed twice with PBS. Finally, the cytotoxic effect of the CD8^+^ T cells on the tumor cells was assessed using the MTT assay.

### 2.22 Statistical analysis

All statistical analyses were performed using R software. Comparison between two groups was conducted using Student’s T-tests or Wilcoxon rank-sum tests. Differences in survival were assessed using log-rank tests. Correlation analyses were performed using Spearman correlation. All statistical tests were two-sided, and differences were considered statistically significant at *P* < 0.05.

## 3 Result

### 3.1 Identification of prognostic palmitoylation- and fatty acid metabolism-related genes in BRCA

**FIGURE 1:**
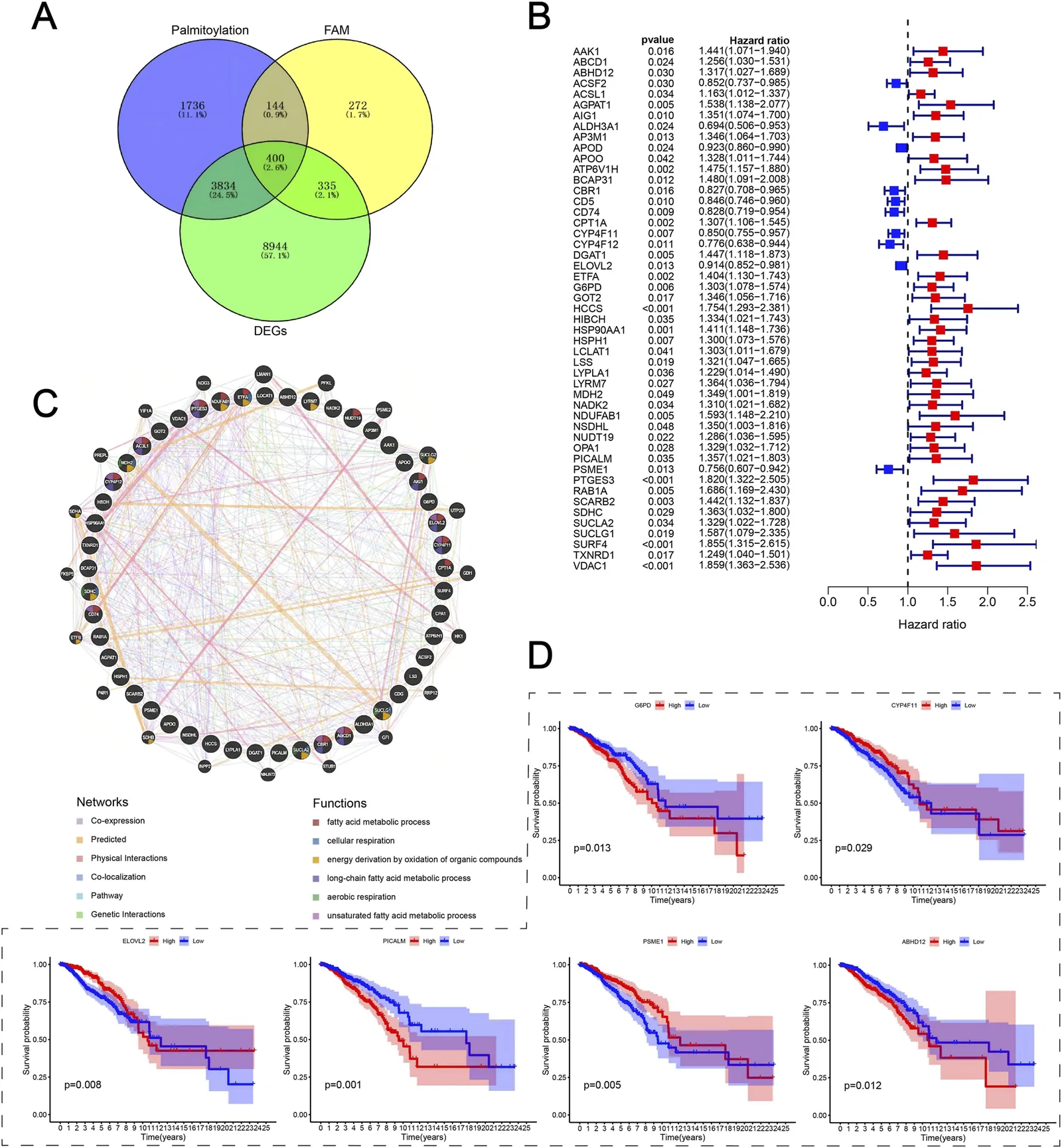
Identification of Prognostic Genes Associated with Fatty Acid Metabolism and Palmitoylation in BRCA. **(A)** A Venn diagram illustrates the intersection among palmitoylation, fatty acid metabolism, and differentially expressed (diff) genes. Genes common to all three categories are highlighted. **(B)** P-values and Hazard Ratios (HR) derived from univariate Cox regression analysis; these genes are identified as having prognostic value in BRCA. Blue indicates low-risk genes, while red indicates high-risk genes. **(C)** Biological network analysis of these predictive genes was performed using GeneMANIA. **(D)** Kaplan-Meier survival curves for selected prognostic genes.

In this study, a combination of gene transcriptional profiles from BRCA patients in the TCGA and GTEx databases was utilized to identify DEGs between BRCA tissues and adjacent normal tissues; a total of 13,513 DEGs were identified. Based on current understanding of metabolic processes, specifically palmitoylation and fatty acid oxidation, 87 fatty acid metabolism-related gene sets were screened from the GSEA database, yielding a curated list of 1,151 fatty acid metabolism genes. Additionally, 6,114 human palmitoylation-related genes were retrieved from the SwissPalm database. By intersecting the sets of DEGs in BRCA, fatty acid metabolism-related genes, and palmitoylation-related genes, a total of 400 genes were identified. These genes, termed PAFARGs, represent differentially expressed genes associated with both palmitoylation and fatty acid metabolism in BRCA. (Figure 1A). Subsequently, univariate Cox regression analysis was performed on these genes to identify those whose expression levels were statistically significantly associated with patients’ survival time; ultimately, 49 prognosis-related PAFARGs were identified. A forest plot was generated based on the results of the univariate Cox regression analysis (Figure 1B). In the forest plot, genes such as ACSF2, ALDH3A1, APOD, and CBR1 are marked in blue, indicating that they act as protective factors with a Hazard Ratio (HR) < 1, thereby favoring a positive prognosis. The remaining genes, including AAK1, are highlighted in red, indicating that they are associated with poor prognosis (HR > 1). In the circular visualization, the genes located in the inner circle represent the identified prognosis-related genes, while the genes in the outer circle represent a predicted set of genes functionally similar or sharing common attributes with the initial prognostic genes. The interactions and functional relationships among these genes are illustrated in Figure 1C. Among these genes, the expression levels of 29 were found to be associated with survival time in BRCA; Figure 1D presents survival-expression analysis plots for a subset of these genes. Finally, we investigated the mutation profiles of these prognosis-related genes. A waterfall plot revealed that the overall mutation frequency across these 49 prognostic genes was 20.88%; individually, the mutation frequencies for most genes were relatively low, with the highest reaching 2% (Supplementary Figure S1B). Copy Number Variation (CNV) analysis of these 49 prognostic genes revealed that CPT1A exhibited the highest frequency of copy number amplification, while SUCLA2 showed the highest frequency of copy number deletion (Supplementary Figure S1A). The chromosomal locations of mutations for these 49 genes across the 23 pairs of chromosomes are illustrated in Supplementary Figure S1C, demonstrating that mutations are present across the majority of chromosomes. These observed differences in copy number variation patterns hold significant implications for investigating gene therapy strategies for BRCA and for identifying potential therapeutic targets.

### 3.2 Pan-cancer analysis of prognostic PAFARGs

**FIGURE 2:**
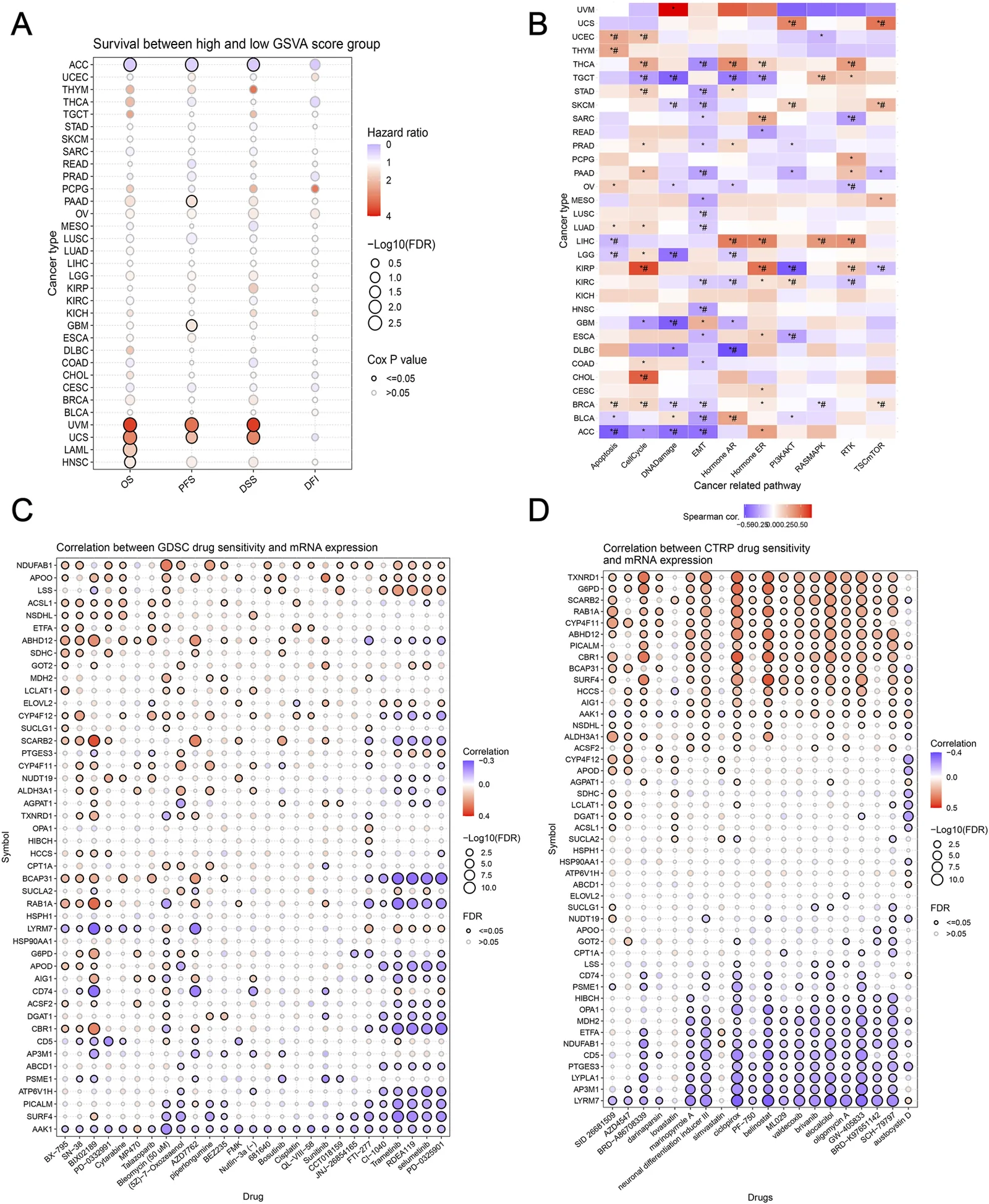
Pan-Cancer Analysis of PAFARGs. **(A)** GSVA scores, calculated based on genes associated with fatty acid metabolism and palmitoylation, illustrating survival differences across various cancer types between high-score and low-score groups. **(B)** Associations between GSVA scores and the activity of cancer-related pathways (*: *p*-value ≤0.05; #: FDR ≤0.05). **(C,D)** Summary of the relationships between gene expression and the efficacy of the top 30 drugs from the GDSC and CTRP databases within the pan-cancer analysis.

In the preceding section, preliminary analyses identified 49 PAFARGs that potentially hold prognostic value for patients with BRCA. To further elucidate the biological functions and clinical significance of these PAFARGs, a pan-cancer analysis was conducted using the Gene Set Cancer Analysis (GSCA) tool. This pan-cancer analysis aimed to identify both the commonalities and specificities of these genes within the context of tumor development, thereby enhancing the broad applicability and generalizability of this study. First, GSCA assessed the associations between GSVA scores, which were derived from the set of 49 genes, and survival outcomes (including OS, PFS, DSS, and DFI) across 33 distinct cancer types. The GSVA score serves as a positive correlate of the collective expression activity of these prognostic genes within a given target tumor type. As illustrated in Figure 2A, these prognostic genes were marked with solid red circles in several cancer types, including UVM (Uveal Melanoma), UCS (Uterine Carcinosarcoma), LAML (Acute Myeloid Leukemia), and HNSC (Head and Neck Squamous Cell Carcinoma), indicating that patient groups with higher GSVA scores experienced poorer survival outcomes compared to those with lower scores. Regarding BRCA, although the observed statistical difference did not reach the threshold for significance, the data nonetheless suggested a potential underlying trend wherein higher GSVA scores for these prognostic genes are associated with poorer survival outcomes in BRCA patients. To further analyze the biological mechanisms of prognostic genes across various cancer types, we investigated the relationship between GSVA scores and the activity of cancer-related pathways. The results indicated that, in the epithelial-mesenchymal transition (EMT) pathway, which was observed in the majority of cancer types, higher GSVA scores correlated with stronger pathway inhibition; this suggests that patients exhibiting higher overall expression levels of these prognostic genes face a lower risk of cancer motility and metastasis. Furthermore, the results demonstrated that, specifically in BRCA, the expression levels of these prognostic genes were significantly correlated with the activity of pathways such as Apoptosis, Cell Cycle, and DNA Damage Response (Figure 2B). Finally, we utilized GSCA to analyze the correlation between the expression of these prognostic genes and sensitivity to drugs listed in the GDSC and CTRP databases (focusing on the top 30 drugs) (Figures 2C,D). Bubble plots revealed that, within the CTRP database, the expression levels of genes such as TXNRD1, G6PD, SCARB2, RAB1A, and CYP4F11 were positively correlated with sensitivity to various CTRP drugs. Conversely, genes such as LYRM7, AP3M1, and LYPLA1 exhibited the opposite correlation patterns. Similarly, we identified additional genes within the GDSC database that demonstrated correlations with various anti-tumor drugs. These findings collectively validate that the prognostic genes identified in this study possess inherent correlations with a diverse range of anti-tumor therapeutic agents.

### 3.3 Unsupervised clustering analysis identifies two prognostic subtypes associated with PAFARGs

**FIGURE 3:**
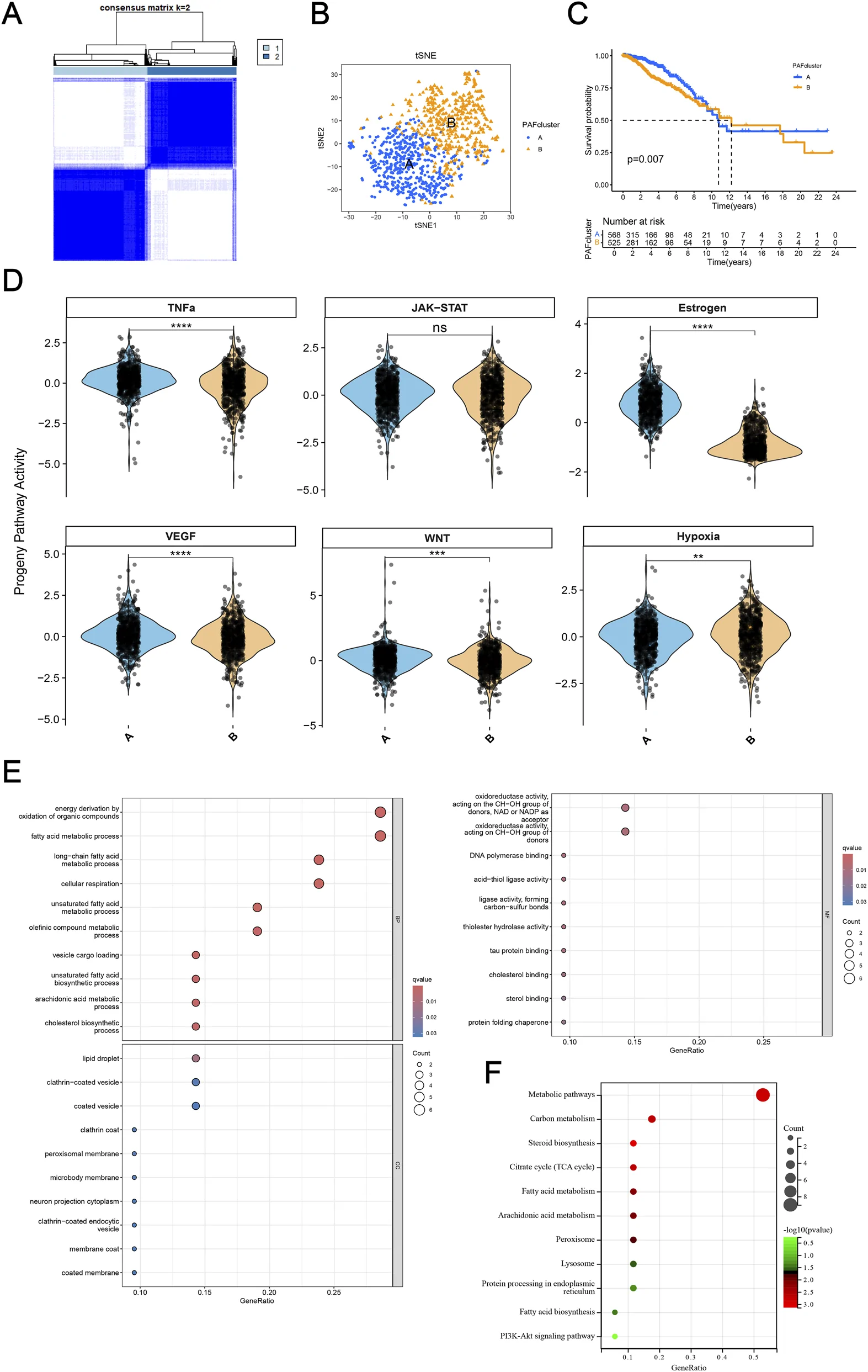
Consensus Clustering Identifies Two Prognostic Subtypes Associated with PAFARGs. **(A)** Consensus clustering analysis identifies two distinct BRCA subtypes characterized by unique biological functional features; K = 2 is determined to be the optimal number of clusters. **(B)** t-SNE visualization demonstrates significant transcriptional differences between the two subtypes, indicating that the two groups are clearly distinguishable. **(C)** Survival analysis based on the two subtypes, revealing significant differences in survival duration between the two groups. **(D)** Assessment of pathway activation status within the two subtypes using PROGENy (Pathway Response Genes for Activity Inference). **(E)** GO enrichment analysis comparing the two subtypes. **(F)** KEGG enrichment analysis comparing the two subtypes.

Consensus clustering analysis was performed based on the gene expression profiles of 49 prognostic PAFARGs to classify BRCA samples. As shown in Figure 3A, the optimal number of clusters was determined to be two; consequently, the subsequent analysis divided all BRCA samples into two distinct subtypes, which a result further validated by t-SNE visualization (Figure 3B). Subsequently, survival analysis was conducted using the “survival” R package to assess differences in overall survival (OS) between the two subtypes; the results revealed significant differences in both survival duration and survival status between the two groups (Figure 3C). To investigate the underlying biological activities distinguishing these two subtypes, which are associated with palmitoylation and fatty acid metabolism, we employed PROGENy (Pathway Response Genes for Activity Inference) to evaluate the activation levels of various cellular signaling pathways within each subtype. Our results indicate differential activation of various cellular signaling pathways, including those involving TNFα, hypoxia-related pathways, and others, whereas no significant difference was observed in JAK-STAT signaling pathways between the two groups (Figure 3D). This result prompted us to conduct further investigations into the biological distinctions between these two groups. Furthermore, we identified DEGs between the two subtypes for downstream analysis (|log2FC| > 0.5 and *p* < 0.05). A heatmap illustrating the expression patterns of these DEGs is presented in Supplementary Figure S2A. Based on this analysis, a total of 22 genes were identified as significantly differentially expressed between the two subtypes (Supplementary Table S1). We then subjected these DEGs to GO and KEGG functional enrichment analyses. The GO enrichment analysis indicated that these DEGs were primarily enriched in processes such as “energy derivation by oxidation of organic compounds” and “fatty acid metabolic processes” pathways, directly involved in fatty acid metabolism (Figure 3E). The KEGG enrichment analysis revealed that these DEGs are predominantly involved in the regulation of signaling pathways such as “Metabolic pathways” and “Carbon metabolism” (Figure 3F). Collectively, these results further suggest that the core drivers of palmitoylation may be intricately linked to multiple downstream oncogenic signaling pathways.

### 3.4 Construction and Validation of a prognostic model associated with PAFARGs in BRCA patients

**FIGURE 4:**
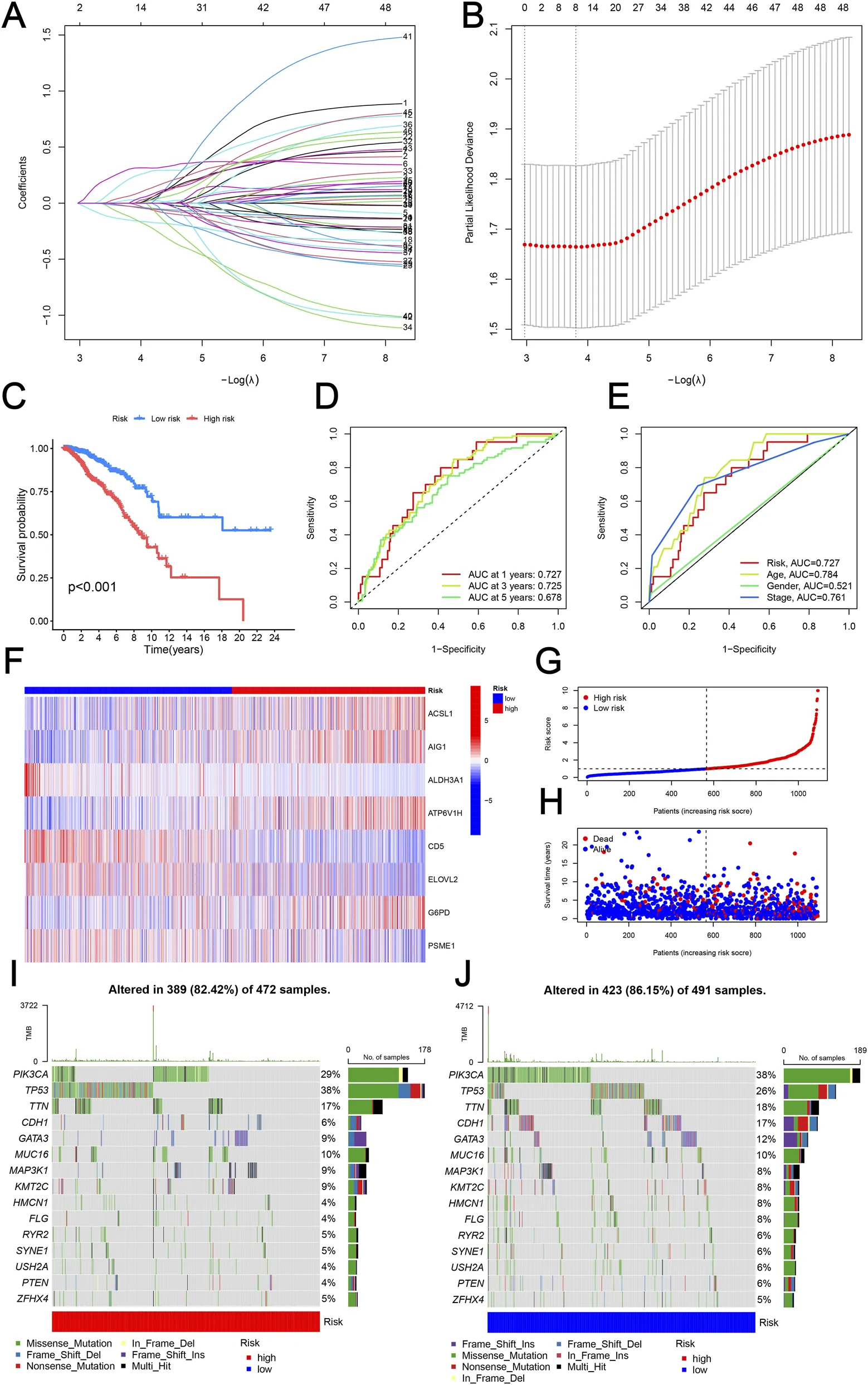
Construction and Validation of a Prognostic Model Based on PAFARGs in BRCA. **(A,B)** Identification of signature genes and construction of a prognostic model for BRCA patients using multivariate Cox regression and LASSO Cox regression .**(C)** Kaplan-Meier survival curves illustrating the difference in overall survival (OS) between the two groups; the low-risk group demonstrates significantly better OS than the high-risk group. **(D)** ROC curves predicting the sensitivity and specificity of the risk score model for 1-, 3-, and 5-year survival rates. **(E)** Time-dependent ROC analysis demonstrating the predictive power of the risk signature and other clinical features. **(F)** Heatmap illustrates the differential expression of risk-associated genes between the low- and high-risk score groups. **(G)** Distribution of patients across the risk score spectrum for both groups. **(H)** Survival status of patients within the different risk groups; red dots represent deceased patients, while blue dots represent surviving patients. **(I,J)** Gene mutation profiles comparing the low-risk and high-risk groups.

Based on the aforementioned analyses, this study confirmed that the identified genes associated with PAFARGs hold significant value as prognostic markers for BRCA patients, thereby demonstrating the feasibility of utilizing these genes to construct a prognostic model. LASSO regression combined with multivariate Cox regression was employed to screen for key genes to construct a proportional hazards regression model for patient prognosis. Through identification via LASSO-Cox regression in both the training and testing sets, genes exhibiting the most favorable multivariate outcomes and the highest predictive values were selected to construct the model (Figures 4A,B). Ultimately, eight genes (ACSL1, AIG1, ALDH3A1, ATP6V1H, CD5, ELOVL2, G6PD, and PSME1) were selected to construct the prognostic risk model. The risk scores for each gene are presented in Supplementary Table S2. Utilizing the expression levels of each gene and the coefficients derived from the LASSO regression analysis, a risk score was calculated for each sample. Based on the median risk score, all samples were stratified into a low-risk group and a high-risk group. Survival curves demonstrated that the group with higher risk scores experienced poorer prognostic outcomes (Figure 4C), thereby validating the practical significance of risk stratification for the prognosis of BRCA patients. ROC curves were utilized to assess the predictive capability and accuracy of the model; a larger Area Under the Curve (AUC) indicates stronger potential predictive power of the risk model for BRCA patients, with a value greater than 0.6 signifying a positive predictive result. The AUC values for patient survival at 1, 3, and 5 years were 0.727, 0.725, and 0.678, respectively (Figure 4D). Furthermore, ROC curves incorporating clinical features demonstrated that, when compared with other clinical characteristics, the risk score exhibited a comparably high AUC value (AUC = 0.727) in terms of predictive power (Figure 4E). These results indicate that the constructed model is capable of reliably predicting patient survival. A heatmap was generated (Figure 4F) to visualize the differential expression of the eight key genes between the high- and low-risk groups. The results revealed that four genes (ACSL1, G6PD, AIG1, and ATP6V1H) were highly expressed in the high-risk group, whereas the other four genes (ALDH3A1, CD5, ELOVL2, and PSME1) were lowly expressed in the high-risk group; these differential expression patterns potentially underlie the increased prognostic risk observed in BRCA patients. Figures 4G,H illustrate the distribution of risk scores and the corresponding survival status of the patients. Subsequently, a waterfall plot was employed to visualize the gene mutation profiles within the high- and low-risk groups. Analysis of the waterfall plot results reveals that the low-risk group exhibits a higher mutational burden and greater sensitivity to immunotherapy, making them more suitable candidates for treatment with immunotherapeutic agents among BRCA patients (Figures 4I,J). Specifically, classic BRCA-associated genes, such as PIK3CA, TP53, CDH1, and GATA3, demonstrate significant differences in mutation frequencies between the two groups. A focused analysis of PIK3CA mutations, in particular, shows that their frequency in the high-risk group (29%) is significantly lower than in the low-risk group (38%) (Figures 4I,J). This suggests that, in the low-risk group, tumor cells, which are driven by mutations in this specific gene, may possess enhanced growth and anti-apoptotic capabilities, thereby exhibiting more pronounced resistance to treatment. Overall, the results presented in the waterfall plot provide valuable targeted insights for the treatment and prognosis of BRCA patients.

### 3.5 Development of the predictive nomogram

**FIGURE 5:**
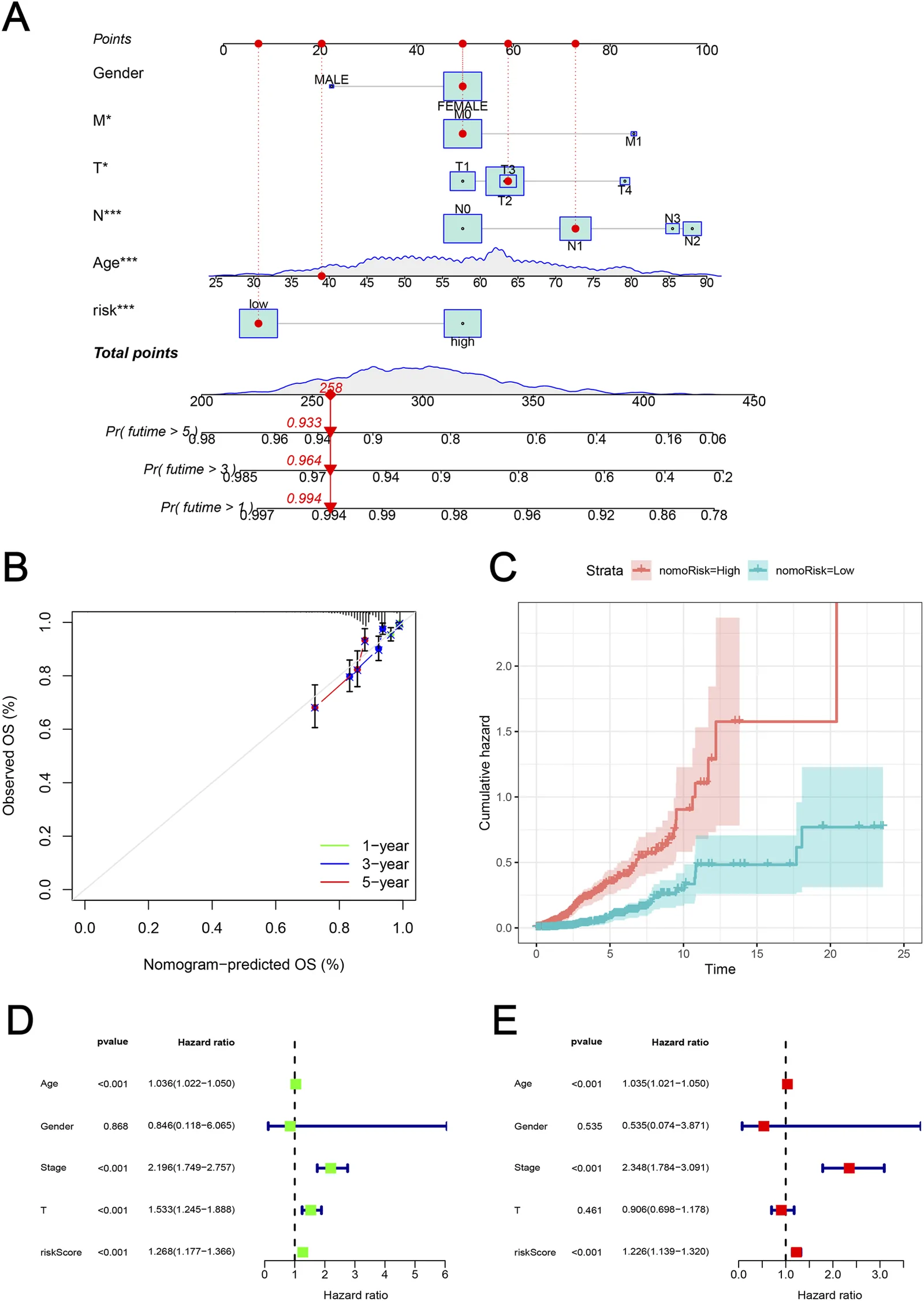
Construction of a Nomogram. **(A)** Construction of a nomogram by integrating the risk score with clinical features to calculate a total patient score and estimate the probability of patient survival. **(B)** Calibration curves assessing the agreement between the constructed model’s predictions and actual observed outcomes. **(C)** Changes in risk over time compared to the low- and high-risk nomogram groups. **(D,E)** Univariate and multivariate Cox regression analyses combining the risk score with clinical features; factors with *P* < 0.05 are identified as independent prognostic factors.

Subsequently, to further validate the accuracy of the constructed model, we developed a nomogram by integrating various clinical parameters (Dong et al., 2024; Xia et al., 2025). Each clinical feature was assigned a specific score; by aggregating the scores for all features, we could estimate a patient’s overall survival rates at the 1-, 3-, and 5-year marks. As shown in Figure 5A, the patient in this example had a total score of 258, corresponding to estimated overall survival probabilities of 0.994, 0.964, and 0.933 at 1, 3, and 5 years, respectively, indicating a favorable prognosis. The accompanying calibration curves further demonstrate that the predictive model’s estimates closely align with actual observed outcomes, thereby confirming the capability of our constructed risk model to accurately predict survival rates in BRCA patients (Figure 5B). Over time, the cumulative risk for patients increases, with the rate of increase in the high-risk group being significantly steeper than that in the low-risk group (Figure 5C). Finally, both univariate and multivariate Cox regression analyses identified age, disease stage, and the risk score as independent prognostic factors for BRCA patients (Figures 5D,E), thereby providing further validation of the reliability of the risk score.

### 3.6 Analysis of the immune microenvironment and prediction of drug treatment response

**FIGURE 6:**
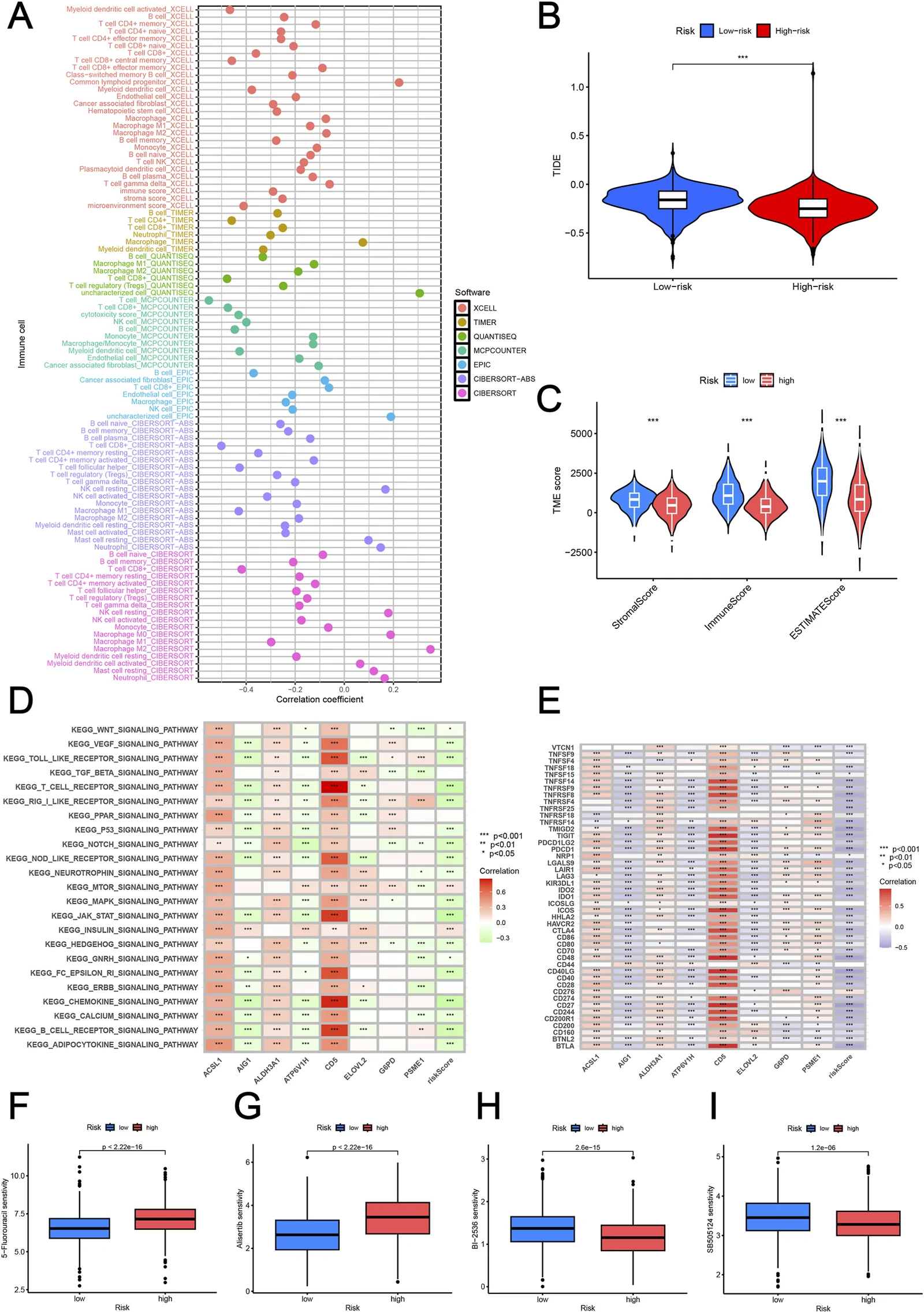
Immune Microenvironment Landscape and Functional Analysis. **(A)** Analysis of the correlation between the risk score and the abundance of various immune cell types using multiple immune cell deconvolution methods. **(B)** Assessment of the potential efficacy of immunotherapy in the low-risk versus high-risk groups. **(C)** Assessment of the differences in TME scores between the low- and high-risk groups. **(D)** Correlation analysis between the risk genes/risk score and KEGG signaling pathways. **(E)** Correlation analysis between the risk genes/risk score and genes associated with immune evasion. **(F,G)** Representative potential therapeutic drugs demonstrating high sensitivity in the high-risk group. **(H,I)** Representative potential therapeutic drugs demonstrating high sensitivity in the low-risk group.

Subsequently, this study further explored the biological basis of the risk model from the perspective of the immune microenvironment. We first applied various algorithms for estimating immune cell infiltration to systematically analyze the correlations between risk scores and the abundance of different immune cell types within the TME, aiming to elucidate the intrinsic links between the risk model and immune regulation (Figure 6A). The results revealed a negative correlation between risk scores and the majority of infiltrating immune cells, suggesting that patients with higher risk scores are more likely to exhibit severe immunosuppression. Subsequently, we employed the Tumor Immune Dysfunction and Exclusion (TIDE) algorithm to investigate the immunotherapy response in BRCA patients belonging to the low-risk and high-risk groups. Our results indicated that there were indeed differences in immunotherapy responses between the two groups, with patients in the low-risk group demonstrating a higher response rate to immunotherapy (Figure 6B). Concurrently, using the ESTIMATE algorithm, violin plots (Figure 6C) were generated to compare differences in TME scores between tumor samples from the high-risk and low-risk groups; this analysis revealed that the stromal, immune, and ESTIMATE scores in the low-risk group were all significantly (*p* < 0.001) higher than those in the high-risk group, indicating that the TME in the low-risk group is richer in stromal components and immune cells, rendering these patients more sensitive to immunotherapy. Furthermore, through Gene Set Variation Analysis (GSVA), we investigated the associations between risk scores and KEGG signaling pathways, finding that risk scores were negatively correlated with the majority of these signaling pathways (Figure 6D). Additionally, we assessed the associations between risk scores and genes related to immune checkpoints; Figure 6E illustrates that the risk scores calculated by our prognostic model exhibited a negative correlation with most genes associated with immune evasion, whereas CD5 demonstrated a significant positive correlation with the majority of immune checkpoint-related genes. All these findings suggest that the prognostic model serves as an effective tool for predicting immunotherapy responses in BRCA patients. Subsequently, we predicted the clinical drug sensitivity of BRCA patients across the high-risk and low-risk groups. Our results identified a total of 164 drugs for which sensitivity differed significantly between the high-risk and low-risk groups, seeing Supplementary Table S3 for details. Figures 6F,G present two representative drugs that demonstrated high sensitivity in the high-risk group, while Figures 6H,I present two representative drugs that demonstrated high sensitivity in the low-risk group. These differences in sensitivity across different groups offer a potential strategy for identifying more effective therapeutic agents for patients with varying risk profiles.

### 3.7 G6PD identified and validated as a key gene

**FIGURE 7:**
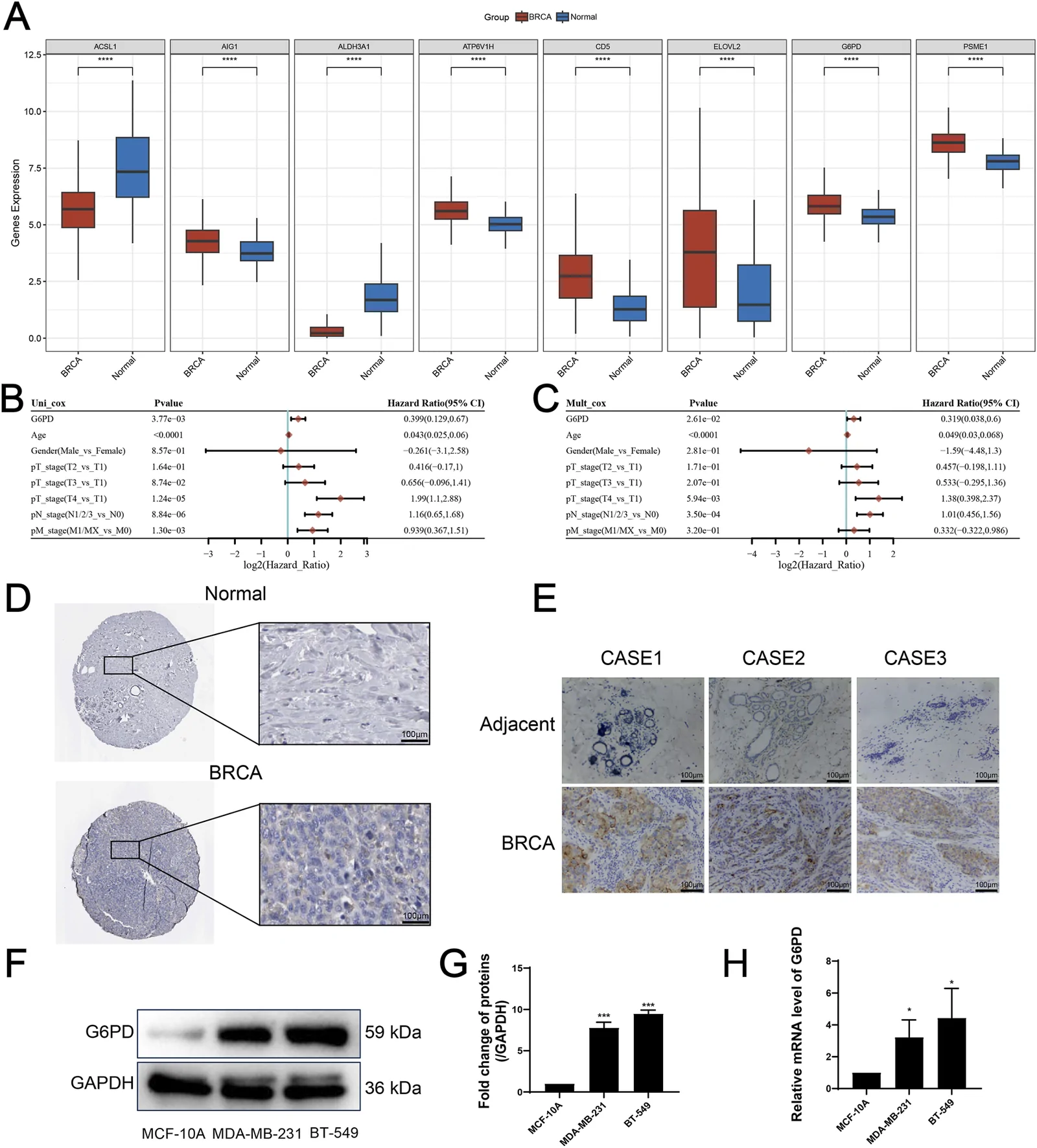
G6PD as an Independent Prognostic Gene Highly Expressed in BRCA. **(A)** Identification of differential expression of risk genes between BRCA tumor samples and normal tissue samples. **(B,C)** Univariate and multivariate Cox regression analyses combining G6PD expression with clinical features demonstrate that G6PD serves as an independent prognostic gene. **(D)** Validation of G6PD expression using the HPA database. **(E)** Immunohistochemical staining illustrating G6PD expression in normal tissues versus BRCA tissues. **(F,G)** Western blot results validating G6PD expression in a normal breast epithelial cell line (MCF-10A) and two invasive BRCA cell lines (BT-549 and MDA-MB-231). **(H)** qPCR further validates G6PD mRNA expression levels in the normal breast epithelial cell line (MCF-10A) and the two invasive BRCA cell lines (BT-549 and MDA-MB-231).

Subsequently, we evaluated the expression patterns of the eight risk-associated genes in both BRCA and normal breast tissues. As shown in Figure 7A, with the exception of ALDH3A1 and ACSL1, which were downregulated in BRCA tissues, the remaining six genes were significantly upregulated in tumor samples. To further clarify the clinical relevance of these genes, we integrated gene expression profiles with survival analysis from our previous results. Among the eight candidates, G6PD consistently exhibited the most robust and stable association with unfavorable overall survival, and patients with high G6PD expression showed a significantly poorer prognosis compared with those with low expression, suggesting its potential oncogenic role. To determine its prognostic independence, we further performed univariate and multivariate Cox regression analyses incorporating G6PD expression and clinicopathological variables (including age, sex, and T/N/M staging). The results demonstrated that G6PD remained statistically significant in both models (*p* < 0.05), indicating that it may serve as an independent prognostic factor (Figures 7B,C). In addition, external validation using the Human Protein Atlas (HPA) database confirmed elevated protein expression of G6PD in BRCA tissues (Figure 7D). Consistently, experimental validation through immunohistochemistry, Western blotting, and qRT-PCR further demonstrated that G6PD is markedly upregulated in both BRCA tissue samples and BRCA cell lines (Figures 7E-H). Importantly, when considering the combined evidence from differential expression, survival association, and multivariate Cox regression analysis, G6PD emerged as the most representative and biologically consistent candidate within the eight-gene signature. Therefore, we selected G6PD as a key focus for subsequent mechanistic investigation to provide biological interpretability for the constructed prognostic model.

### 3.8 Characterization of G6PD via single-cell RNA sequencing analysis

**FIGURE 8:**
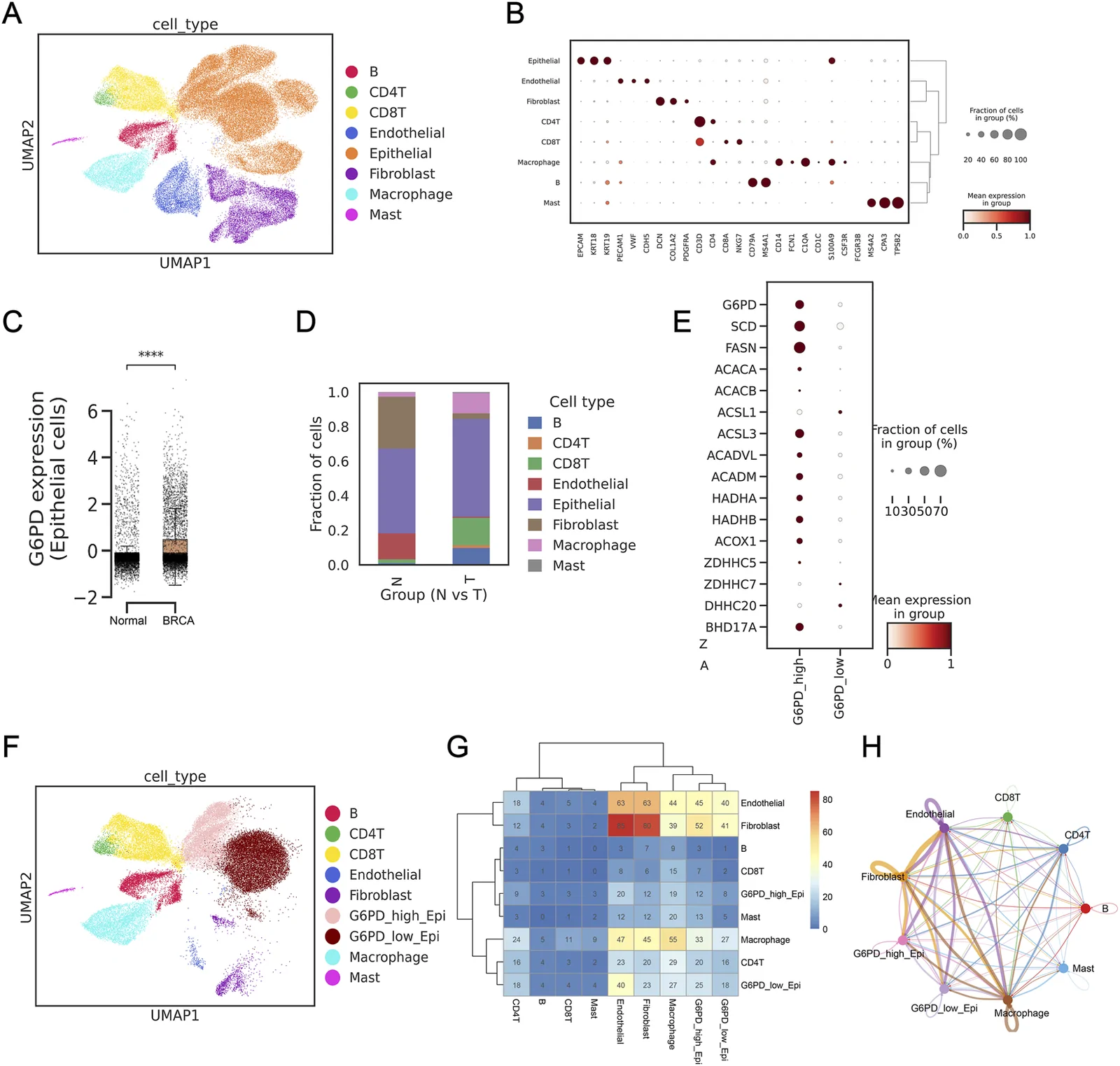
Assessment of Characteristic Genes via Single-Cell Analysis. **(A,B)** Identification of major cell types through sample annotation. **(C)** Upregulation of G6PD in tumor samples compared to normal samples. **(D)** Fractional abundance of different cell types in BRCA versus normal samples. **(E)** The impact of G6PD expression levels on key markers associated with palmitoylation and fatty acid metabolism. **(F)** Redefinition of cell distribution based on G6PD expression levels. **(G,H)** Analysis of differential cell-cell communication mechanisms between G6PD-high epithelial cell populations and G6PD-low epithelial cell populations within the TME using CellChat.

To further elucidate the characteristics of G6PD, we conducted a single-cell analysis using the GSE161529 dataset. From this dataset, we selected six samples of adjacent normal tissue and six samples of BRCA tissue for analysis. We began by performing global cell-type annotation on the dataset, identifying eight major cell types: B cells, CD4^+^ T cells, CD8^+^ T cells, endothelial cells, epithelial cells, fibroblasts, macrophages, and mast cells (Figures 8A,B; Supplementary Figure S2B,C). Subsequently, a comparison between normal and tumor samples revealed that, relative to normal samples, G6PD expression was upregulated in tumor samples (Figure 8C); furthermore, the proportions of fibroblasts and endothelial cells decreased, while the proportion of immune cells increased significantly (Figure 8D). To further validate the role of G6PD in BRCA, we isolated a subset of six tumor samples and classified their epithelial cells into two groups (G6PD_high_epi and G6PD_low_epi) based on their respective G6PD expression levels (Figure 8F). We selected key markers associated with palmitoylation and fatty acid metabolism and observed that the expression levels of these markers were higher in the G6PD_high_epi group compared to the G6PD_low_epi group (Figure 8E). This suggests that the G6PD_high_epi group may exhibit heightened activity in palmitoylation and lipid metabolism, thereby indicating that high G6PD expression likely plays a pivotal role in cellular metabolic reprogramming, particularly within the context of palmitoylation and fatty acid metabolism within the TME. Finally, utilizing CellChat technology, we analyzed the differential cellular communication mechanisms between the G6PD_high_epi and G6PD_low_epi cell populations within the TME. We discovered that the G6PD_high_epi cell population engages in significant interactions with other cell populations (such as endothelial cells, macrophages, and fibroblasts), thereby occupying a central position within the cellular communication network (Figures 8G,H). Collectively, these results suggest that epithelial cells exhibiting high G6PD expression may promote tumor growth and metastasis within the TME through cellular communication.

### 3.9 G6PD knockdown inhibits BRCA cell proliferation, colony formation, and migration in vitro

**FIGURE 9:**
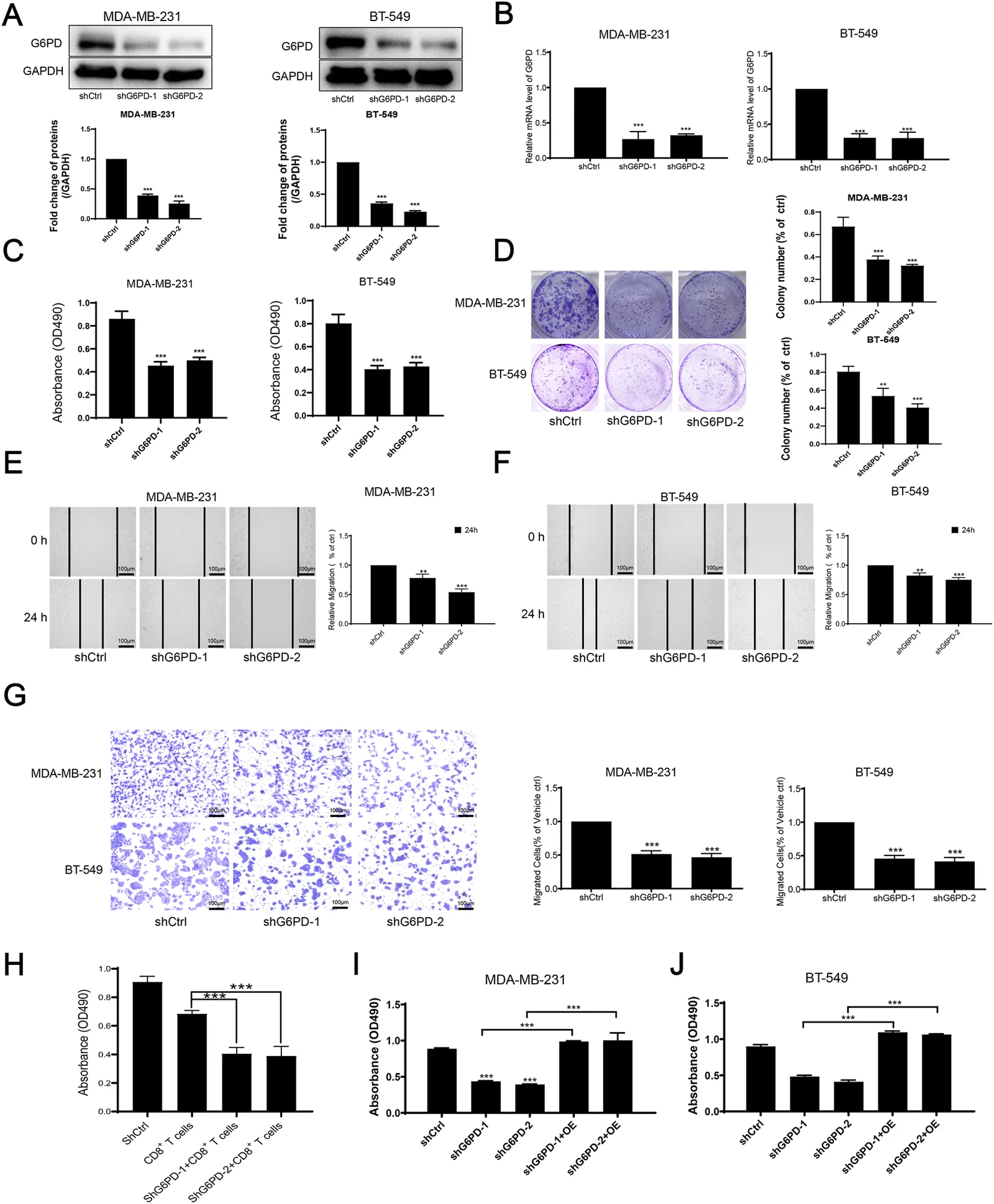
G6PD Knockdown Inhibits the Proliferation and Migration Capabilities of BRCA Cells. **(A)** Western blot validation of G6PD protein expression levels in the BT-549 and MDA-MB-231 cell lines following G6PD knockdown. **(B)** qPCR validation of G6PD mRNA expression levels in the BT-549 and MDA-MB-231 cell lines following G6PD knockdown. **(C)** Assessment of the impact of G6PD knockdown on cell proliferation using the MTT assay. **(D)** Assessment of the impact of G6PD knockdown on colony formation in the BT-549 and MDA-MB-231 cell lines using a colony formation assay. **(E,F)** Wound healing assays assessing the migration capabilities of the two cell lines into the scratched area following G6PD knockdown. **(G)** Transwell chamber assays were performed to assess the migration ability of two cell lines toward the lower chamber following G6PD knockdown. **(H)** MTT assays demonstrated that G6PD knockdown enhances immune cell activity and inhibits the proliferation of BRCA cells when co-cultured with immune cells. **(I,J)** The MTT assay was used to evaluate the reversal of G6PD knockdown-induced inhibition of cell proliferation by G6PD re-expression.

To further validate the impact of G6PD expression levels on the biological behaviors of BRCA cells, specifically proliferation and migration, we utilized shRNA technology to knock down G6PD expression. As shown in Figures 9A,B, sh-G6PD significantly reduced G6PD expression levels. MTT assays demonstrated that G6PD knockdown significantly inhibited the proliferation of BT-549 and MDA-MB-231 cells. The absorbance values (OD values) in the G6PD knockdown group were significantly lower than those in the control group, indicating that G6PD knockdown diminished the proliferative capacity of the tumor cells (Figure 9C). Following G6PD knockdown, the colony-forming abilities of BT-549 and MDA-MB-231 cells decreased significantly (Figure 9D). Wound healing assays revealed that G6PD knockdown significantly inhibited the migratory capabilities of BT-549 and MDA-MB-231 cells (Figures 9E,F). Furthermore, Transwell migration assays confirmed that the migratory potential of BT-549 and MDA-MB-231 cells was significantly reduced following G6PD knockdown (Figure 9G). Finally, we conducted immune cell co-culture experiments to investigate the effect of G6PD knockdown on the proliferation of BT-549 and MDA-MB-231 cells when co-cultured with CD8^+^ T cells. The results indicated that, in the presence of immune cells, the proliferative capacity of tumor cells in the G6PD knockdown group was significantly reduced (Figure 9H). Notably, we performed *in vitro* rescue assays via MTT analysis and found that re-expression of G6PD could reverse the suppression of cell proliferation induced by G6PD knockdown, which further demonstrates the essential role of G6PD in the progression of BRCA (Figures 9I,J). In summary, G6PD knockdown significantly inhibits the proliferation, colony formation, and migratory capabilities of BRCA cells. Moreover, G6PD knockdown further suppresses the proliferation of BRCA cells during co-culture with immune cells by enhancing immune cell activity. G6PD may promote the progression of BRCA by modulating both the biological behaviors of tumor cells and the immune microenvironment. Consequently, G6PD may serve as a potential therapeutic target for BRCA treatment.

### 3.10 G6PD knockdown inhibits BRCA tumor growth in vivo

**FIGURE 10:**
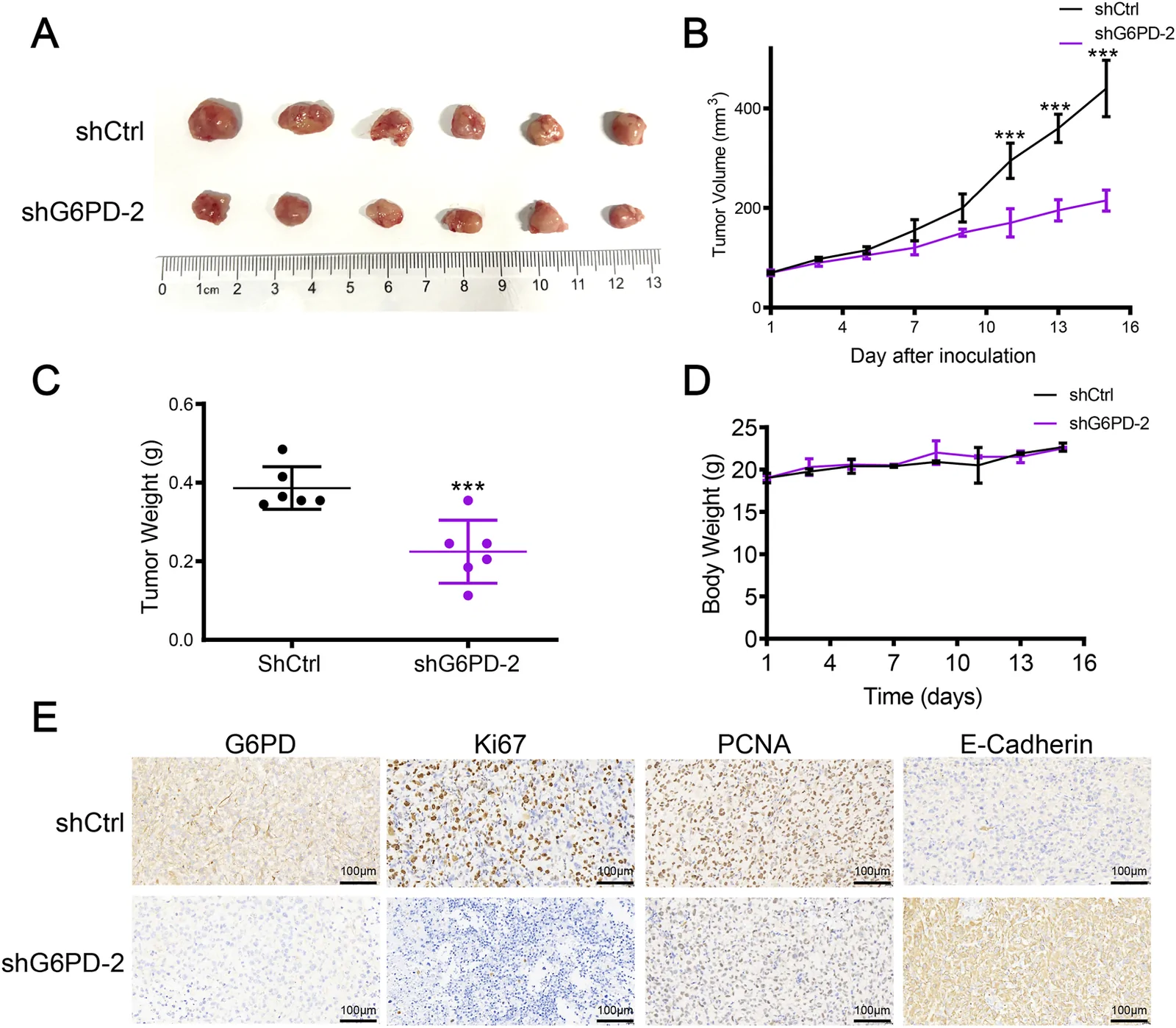
G6PD Knockdown Inhibits Tumor Growth In Nude Mouse Xenograft Model. **(A,B)** Suspensions containing 2 × 10^6^ MDA-MB-231-shCtrl cells and 2 × 10^6^ MDA-MB-231-shG6PD cells were injected into the right flank of mice. Once tumor volumes reached 70–100 mm^3^, the tumor volume of each mouse was measured every 2 days. After 15 days, the tumors were excised and photographed. **(C)** Statistical analysis of tumor weights. **(D)** Changes in body weight for each group of mice over the course of the experiment. **(E)** Immunohistochemistry was used to detect the expression levels of G6PD, Ki67, PCNA, and E-cadherin within the tumor tissues.

We validated the impact of G6PD knockdown on BRCA tumor growth *in vivo* through xenograft tumor formation experiments in nude mice. The results demonstrated that the tumor volume in the G6PD knockdown group was significantly smaller than that in the control group, indicating that G6PD knockdown significantly inhibited tumor growth (Figure 10A). Over time, the rate of tumor volume increase in the G6PD knockdown group was significantly lower than that in the control group; particularly on days 10 and 13, the tumor volume in the G6PD knockdown group was markedly smaller than that in the control group, and the difference was statistically significant (Figure 10B). This further validated that G6PD knockdown effectively inhibits tumor growth. Measurements of tumor weight also supported this conclusion, as the tumor weight in the G6PD knockdown group was significantly lower than that in the control group (Figure 10C). However, G6PD knockdown had no significant effect on the body weight of the mice (Figure 10D). Immunohistochemical staining analysis revealed that G6PD knockdown significantly suppressed the expression of proliferation-associated proteins, Ki67 and PCNA, while simultaneously enhancing the expression of the epithelial cell adhesion protein, E-Cadherin (Figure 10E). These results suggest that G6PD knockdown inhibits the proliferation of BRCA cells by suppressing the expression of Ki67 and PCNA, and may simultaneously suppress the invasiveness of BRCA by enhancing the expression of E-Cadherin.

### 3.11 Drug sensitivity and molecular docking

**FIGURE 11:**
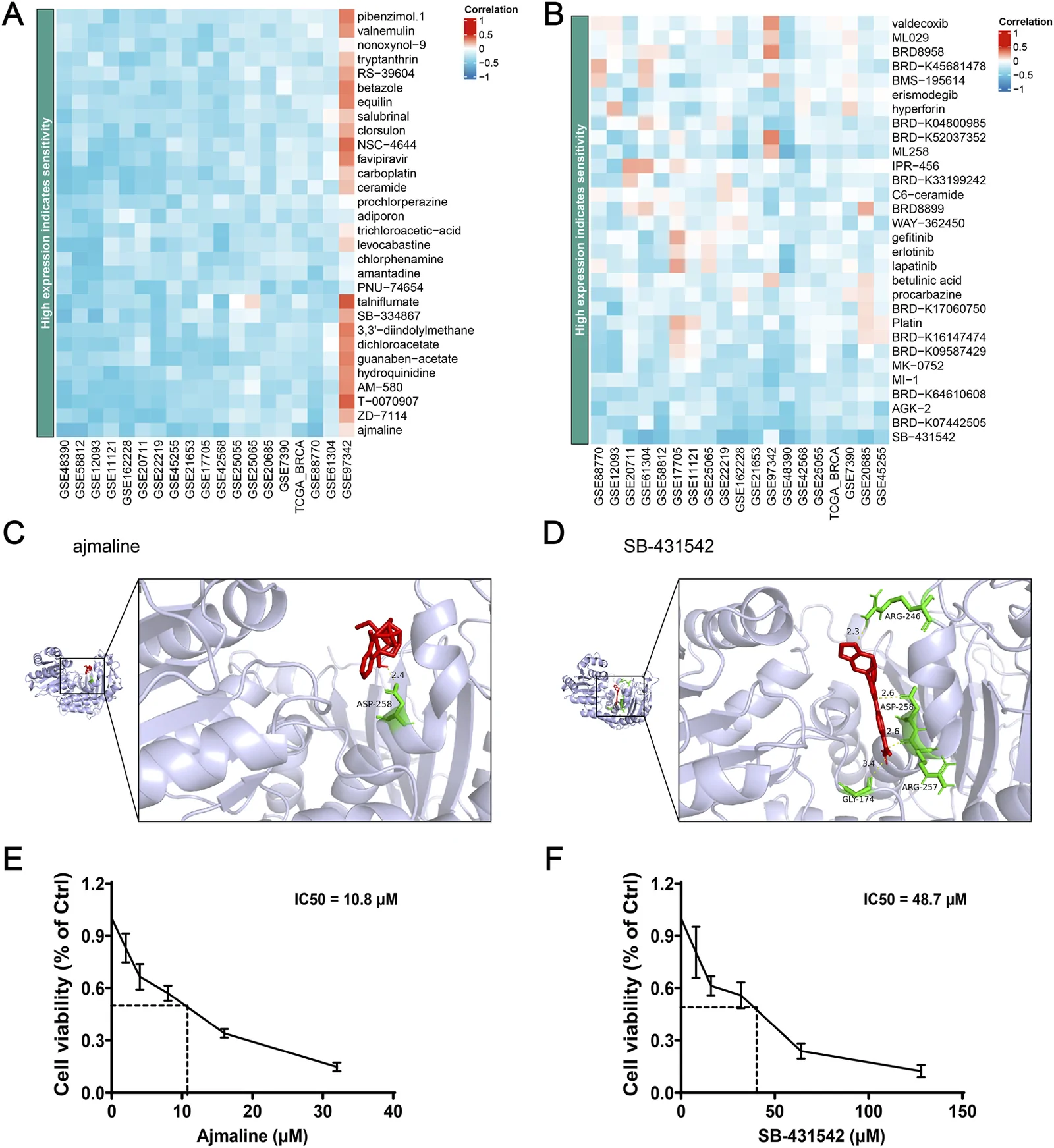
Correlation Analysis of Drug Sensitivity and G6PD Expression, and Molecular Docking. **(A,B)** The BEST tool was utilized to analyze the correlation between G6PD expression and drug sensitivity. **(C,D)** Molecular docking models illustrating the interaction between G6PD and two representative drugs: ajmaline (binding energy: −7.5 kcal/mol) and SB-431542 (binding energy: −8.8 kcal/mol). **(E)** Validation of the inhibitory effect of Ajmaline on BRCA cells via MTT assay. **(F)** Validation of the inhibitory effect of SB-431542 on BRCA cells via MTT assay.

To further investigate targeted therapeutic strategies associated with G6PD, we utilized the BEST database to screen multiple BRCA datasets for small-molecule anti-tumor drugs whose efficacy was significantly correlated with G6PD gene expression levels (Figures 11A,B). Through this analysis, we further identified therapeutic candidate compounds that may be specifically regulated by G6PD. Based on evidence from two independent databases (CTRP and PRISM), we selected two drugs that exhibited a significant negative correlation with high G6PD expression: ajmaline and SB-431542. Subsequently, molecular docking techniques were employed to evaluate the binding affinity between these two drugs and the G6PD protein. The docking results indicated that the binding energy for ajmaline was −7.5 kcal/mol, while that for SB-431542 was −8.8 kcal/mol (Figures 11C,D), indicating strong binding interactions for both compounds. We further performed MTT assays to verify the anti-tumor activity of these two compounds, and the results revealed that both ajmaline and SB-431542 exerted potent anti-tumor effects in BRCA cells (Figures 11E,F). These data suggest that these two compounds may effectively interact with the active site of G6PD, thereby potentially inhibiting its kinase function or interfering with its pro-tumorigenic role in tumor cells.

## 4 Discussion

From the perspective of lipid metabolism reprogramming, this study systematically elucidates the significant value of PAFARGs in the prognostic stratification of BRCA and the prediction of immunotherapy response. Through multi-cohort integrative analysis and machine learning modeling, we constructed prognostic signatures capable of robustly distinguishing patients with different survival outcomes, thereby demonstrating the high explanatory power of lipid metabolism-related molecular features in characterizing the biological heterogeneity of BRCA.

Unlike previous studies that primarily focused on single metabolic pathways or individual metabolic enzymes, this study emphasizes the functional coupling relationship between palmitoylation modification and fatty acid metabolism. Palmitoylation is a post-translational modification found in G protein-coupled receptors that influences signal transduction by regulating protein localization and stability (Qanbar and Bouvier, 2003). Fatty acid metabolism provides the essential palmitic acid substrate required for palmitoylation; in tumor cells, where fatty acid synthesis and uptake are significantly enhanced, the intracellular pool of palmitic acid expands, thereby driving the palmitoylation of numerous key proteins (Du et al., 2026; Buss and Sefton, 1986). Palmitoylation can enhance the stability and localization efficiency of proteins within cell membranes or signaling complexes, enabling the sustained activation of multiple pro-tumor signaling pathways, such as the PI3K/AKT and MAPK pathways (Sun et al., 2022; Guo et al., 2025; Summers et al., 2018). These pathways, in turn, promote fatty acid synthesis, the maintenance of redox homeostasis, and metabolic adaptation, further amplifying lipid metabolism reprogramming (Li et al., 2015; Sun et al., 2023). This positive feedback loop endows tumor cells with a sustained proliferative and invasive advantage under conditions of high lipid supply and strong signaling drive. Furthermore, palmitoylation can regulate the secretion of intercellular signaling molecules and receptor activity, influencing the functional states of immune and stromal cells within the TME, thereby facilitating immune evasion and tumor progression (Du et al., 2021). Consequently, the synergistic dysregulation of palmitoylation and fatty acid metabolism serves not only as a hallmark of tumor metabolic reprogramming but also offers potential entry points for therapeutic intervention targeting the metabolism-signaling coupling network.

Analyses of the immune microenvironment further support these observations. Associations were observed between risk scores and immune cell infiltration, immune checkpoint gene expression, and predicted immunotherapy response, suggesting that lipid metabolic status may be involved in regulating anti-tumor immune responses through modulation of the TME. Previous studies have reported that dysregulated fatty acid metabolism can affect immune cell function and contribute to immunosuppressive states (Luo et al., 2022; Huang et al., 2025). Building on this evidence, the present study provides multi-gene signature–based insights into the relationship between metabolism and immunity, and offers a potential framework for interpreting heterogeneity in immunotherapy response among BRCA patients.

At the level of key individual genes, we further identified G6PD as a central metabolic factor with independent prognostic significance. G6PD is a rate-limiting enzyme in the pentose phosphate pathway (PPP), catalyzing the production of NADPH, which is essential for maintaining redox homeostasis and supporting anabolic biosynthesis in rapidly proliferating cells (Shah et al., 2024). Consistently, rapidly proliferating tumor cells rely on enhanced PPP activity to meet increased demands for nucleotide synthesis and oxidative stress adaptation (Meng et al., 2022). In line with these observations, G6PD expression was significantly upregulated in BRCA in this study, which may reflect elevated metabolic requirements associated with tumor progression. Previous studies have reported that aberrant activation of G6PD is associated with poor prognosis in multiple malignancies, including non-small cell lung cancer and low-grade glioma (Jiang et al., 2021; Yang et al., 2018). Consistently, our results demonstrated that high G6PD expression was significantly associated with unfavorable clinical outcomes in BRCA patients. Functionally, G6PD knockdown significantly suppressed BRCA cell proliferation and migration *in vitro* and reduced tumor growth *in vivo*, further supporting its biological relevance in tumor progression. Notably, single-cell analysis suggested that epithelial cells with high G6PD expression exhibited increased activity in palmitoylation-related processes and fatty acid metabolism, accompanied by altered intercellular communication within the tumor microenvironment. These findings indicate a potential association between metabolic reprogramming and microenvironmental remodeling; however, the underlying molecular mechanisms linking G6PD to lipid metabolic pathways and post-translational modifications remain to be further elucidated. From an immunometabolic perspective, PPP-derived NADPH plays a critical role in supporting reactive oxygen species (ROS) production in immune effector cells, which is essential for phagocytic and cytotoxic functions (Shah et al., 2024; Lapenna, 2023). In addition, previous studies have suggested that G6PD is involved in T-cell activation-related signaling processes through regulation of redox balance and metabolic flux (Gu et al., 2021). In this context, the observed correlation between G6PD expression and immune cell infiltration (including macrophages, CD4^+^ T cells, and CD8^+^ T cells) may reflect a broader coupling between tumor metabolic states and immune microenvironment dynamics, rather than a direct regulatory effect. Collectively, these results suggest that G6PD may function as a key metabolic regulator in BRCA, linking glucose metabolism reprogramming with lipid metabolic alterations and tumor microenvironment remodeling. However, its precise mechanistic role in coordinating these processes requires further experimental validation. Meanwhile, G6PD may serve as a potential prognostic biomarker for BRCA, providing a theoretical basis for future investigations into metabolism-targeted therapeutic strategies.

Furthermore, through drug sensitivity analysis and molecular docking, this study explored the potential pharmacological relevance of G6PD in BRCA, suggesting that its expression level may be associated with differential drug response patterns. Among the analyzed compounds, several agents have been clinically applied for BRCA treatment. 5-fluorouracil is a commonly used chemotherapeutic agent; however, its clinical efficacy is often limited by the development of resistance, which may also contribute to increased toxicity and reduced therapeutic benefit (Yi et al., 2020). Alisertib, a small-molecule inhibitor targeting Aurora kinase A, has also been investigated in BRCA therapy, although resistance to targeted and combination regimens, including CDK4/6-based strategies, remains a significant clinical challenge (Haddad et al., 2023). These observations highlight the importance of identifying additional therapeutic candidates, particularly those suitable for combination strategies. In this context, ajmaline, an antiarrhythmic agent (Monasky et al., 2021), was identified through molecular docking analysis to exhibit favorable binding affinity with G6PD, suggesting its potential as a repurposing candidate for further investigation in BRCA. Previous docking studies have also reported an interaction between ajmaline and YAP with a binding affinity of approximately −6.5 kcal/mol (Balavaishnavi et al., 2024), although the functional relevance of this interaction remains to be experimentally validated. Similarly, SB-431542, a TGF-β receptor inhibitor, has been reported to modulate cancer stem cell-related phenotypes and exert anti-tumor effects through both direct tumor cell inhibition and tumor microenvironment regulation in BRCA models (Butkute et al., 2025). These findings suggest its potential value in targeting tumor progression-related pathways. In addition to these compounds, several other candidate agents were identified in this study. Although these results remain computational predictions, they provide a basis for further exploration of metabolism-associated drug sensitivity patterns and may facilitate future integration of metabolic signatures into therapeutic stratification strategies.

It is important to acknowledge that this study has several limitations. First, although we integrated data from multiple public databases and performed cross-validation, the overall analysis was mainly based on retrospective cohorts. Differences in patient demographics, sequencing platforms, and data processing strategies across datasets may introduce a certain degree of heterogeneity, which could affect the robustness and generalizability of the predictive model. Therefore, further validation in independent multicenter prospective cohorts is warranted. Second, this study characterizes palmitoylation-related and fatty acid metabolic features primarily at the transcriptomic level. However, corresponding protein-level validation, metabolic flux analysis, and dynamic profiling of palmitoylation modifications were not performed. As a result, the current findings mainly reflect transcript-level associations rather than fully capturing real-time biological activity. Third, although *in vitro* and *in vivo* experiments have demonstrated the tumor-promoting role of G6PD in BRCA progression, the precise molecular mechanisms by which G6PD regulates immune-related processes and intercellular signaling networks, particularly in the context of palmitoylation and fatty acid metabolism, remain to be further elucidated. This represents an important direction for future mechanistic investigation. Importantly, this study did not include breast cancer patients who had actually received immunotherapy; therefore, the conclusions regarding immunotherapy response are based on computational inference (including TIDE scores and immune infiltration analyses) and should be interpreted as indicative of potential response patterns rather than direct clinical prediction. Future validation using prospective immunotherapy cohorts or real-world clinical datasets will be essential to confirm these findings. Finally, although drug sensitivity analyses and molecular docking results suggest that G6PD may represent a potential therapeutic target, these findings remain exploratory and require further validation in patient-derived models and preclinical systems before clinical translation.

## 5 Conclusion

This study constructed and validated a PAFARG-related prognostic signature in BRCA, suggesting a potential link between lipid metabolic reprogramming and the tumor immune microenvironment. G6PD was identified as a key metabolic gene associated with BRCA progression and tumor biological behavior. These findings provide insights into BRCA metabolic characteristics and suggest possible associations with immune-related features. However, the conclusions regarding immune regulation and clinical implications remain exploratory and require further validation. Overall, this study offers a potential framework for prognostic stratification and highlights metabolic genes as promising targets for future investigation.

## Data Availability

The original contributions presented in the study are included in the article/Supplementary Material, further inquiries can be directed to the corresponding author.
