# Supplementary material for: Gene signatures of palmitoylation and fatty acid metabolism predict prognosis and immunotherapy response in breast cancer patients by machine learning, single-cell analysis, and experimental validation
Source: Front Pharmacol. 2026 Aug 7;17:1856591. doi: 10.3389/fphar.2026.1856591 (PMC13493559; doi:10.3389/fphar.2026.1856591)
Supplement: Supplementary file 1 [file Supplementaryfile1.docx]

Supplementary Material

**Supplementary Figure 1 and Supplementary Tables 1-2**

**Figure S1**

**Figure S1.** (A) The frequency of CNV amplification and deletion of these prognostic genes. (B) The locations of these prognostic genes mutations on 23 pairs of chromosomes. (C) The mutation frequency of these prognostic genes in BRCA patients in the TCGA cohort.

**Figure S2.**

**Figure S2.** (A) Differentially expressed genes in the two subtypes were identified through consensus clustering, with the filtering condition log2(FC)| > 0.5 and *p* < 0.05. (B, C) The t-SNE plot shows the distribution of normal and tumor samples identified from BRCA single-cell RNA sequencing (scRNA-seq) data.

**Supplementary Table 1.** List of 22 DEGs.

| **Gene** | **logFC** | **AveExpr** | **t** | **P.Value** | **adj.P.Val** |
| --- | --- | --- | --- | --- | --- |
| ELOVL2 | -2.332450241 | 3.9189913308972 | -7.568398839 | 1.21402571855266e-11 | 5.94872602090804e-10 |
| APOD | -1.981290678 | 7.71181000774766 | -4.922231427 | 3.01205909144293e-06 | 7.37954477403518e-05 |
| ETFA | -0.631194325 | 6.08882585672897 | -4.780665691 | 5.42391994245318e-06 | 8.8590692393402e-05 |
| ABCD1 | 0.615534395510285 | 4.6040287772243 | 4.61653031136127 | 1.05921287725273e-05 | 0.00012975357746346 |
| SDHC | 0.435751924781913 | 7.63643708517757 | 4.12023388764918 | 7.33774841520753e-05 | 0.000719099344690338 |
| ABHD12 | 0.460793801349997 | 6.32287168548598 | 3.97355903685754 | 0.0001265571770963 | 0.000933750494583134 |
| NSDHL | 0.399425778894678 | 6.06831443753271 | 3.95921786266936 | 0.000133392927797591 | 0.000933750494583134 |
| SCARB2 | -0.439799207 | 6.32972251588785 | -3.708335346 | 0.000328040741698052 | 0.00200924954290057 |
| CBR1 | 0.666379397268791 | 5.39235615635514 | 3.572751408 | 0.000524763866205864 | 0.00267488131027804 |
| LYRM7 | -0.400858367 | 3.88183834020561 | -3.561200704 | 0.000545894144954701 | 0.00267488131027804 |
| BCAP31 | 0.346206321954254 | 8.52751317173832 | 3.33885551733967 | 0.00114736709169236 | 0.00511099886299323 |
| SUCLA2 | -0.358713417 | 4.47935929830841 | -3.108730496 | 0.00238967695848486 | 0.00975784758047983 |
| ACSL1 | -0.63232991 | 6.13590857066355 | -3.070190912 | 0.00269245570570024 | 0.0101484868907163 |
| PTGES3 | -0.280654621 | 8.91159380020561 | -2.758130421 | 0.0068046206340899 | 0.0228726199515591 |
| AP3M1 | -0.354413892 | 5.8585383861215 | -2.748124247 | 0.00700182243415075 | 0.0228726199515591 |
| HSP90AA1 | -0.360260237 | 10.3062425058972 | -2.644052029 | 0.00938357569806899 | 0.0287372005753363 |
| LSS | 0.328706314337586 | 5.20869082735514 | 2.58567665146124 | 0.0110198496942688 | 0.0317630961775984 |
| HCCS | 0.251521791150708 | 5.60322857848598 | 2.50670445128949 | 0.0136415652513643 | 0.0363713473841249 |
| CD5 | 0.585070936665601 | 3.07428847459813 | 2.49423304640895 | 0.0141031755162933 | 0.0363713473841249 |
| PICALM | -0.265428463 | 6.95069124157944 | -2.333011376 | 0.0214574916193607 | 0.048828732931539 |
| G6PD | 0.351388752403544 | 6.10454382173832 | 2.32823092421693 | 0.0217196611099826 | 0.048828732931539 |
| RAB1A | -0.212583353 | 8.03459808476635 | -2.324556247 | 0.0219231045815073 | 0.048828732931539 |

**Supplementary Table 2.** Eight PAFARGs were selected by multivariate Cox results.

| **PAFARGs** | **coef** |
| --- | --- |
| ACSL1 | 0.187494570312718 |
| AIG1 | 0.403649539594543 |
| ALDH3A1 | -0.325341446 |
| ATP6V1H | 0.488341099411207 |
| CD5 | -0.240591313 |
| ELOVL2 | -0.13991874 |
| G6PD | 0.244004100245406 |
| PSME1 | -0.37242066 |

PAFARGs, Differentially expressed Palmitoylation- and Fatty Acid Metabolism-Related Genes; Coef, coefficient.

**Supplementary Table 3.** Detailed IC50-values for the 164 drugs.
